# Prospective Proteomic Associations with Incident Type 2 Diabetes and Responses to Metabolic Stress in Women with and Without Polyendocrine Metabolic Ovarian Syndrome

**DOI:** 10.3390/ijms27219894

**Published:** 2026-11-04

**Authors:** Radwan Darwish, Thozhukat Sathyapalan, Laura Dempsey, Mohammed Altigani Abdalla, Alexandra E. Butler, Stephen L. Atkin

**Affiliations:** 1 School of Medicine, Royal College of Surgeons in Ireland-Medical University of Bahrain (RCSI-MUB), Busaiteen 228, Bahrain; 22200493@rcsi-mub.com; 2 Academic Endocrinology, Diabetes and Metabolism, Hull York Medical School, Hull HU6 7RU, UK; 3 Data Science Centre, School of Population Health, Royal College of Surgeons in Ireland, RCSI, D02 YN77 Dublin, Ireland; 4 School of Postgraduate Studies and Research, Royal College of Surgeons in Ireland-Medical University of Bahrain (RCSI-MUB), Busaiteen 228, Bahrain

**Keywords:** type 2 diabetes, proteomics, adiponectin, polyendocrine metabolic ovarian syndrome, insulin resistance, lipid infusion, metabolic stress

## Abstract

Women with polyendocrine metabolic ovarian syndrome (PMOS) have heterogeneous metabolic risk. We investigated whether targeted circulating proteins were associated with incident type 2 diabetes (T2D) and whether the retained proteins responded to experimentally induced lipid stress. In Cohort 1, 231 women with (*n* = 135) or without (*n* = 96) PMOS had T2D outcome data, of whom 25 developed T2D during a median follow-up of 12.7 years (range 10.2–15.1); 221 women (20 incident cases) had complete covariate data for adjusted analyses. Of 44 available proteins, 27 were prespecified from prior literature; following Benjamini–Hochberg adjustment, eight biomarkers (Adiponectin (ADIPOQ), Endocan (ESM1), Mast/stem cell growth factor receptor-Kit (KIT), Chordin Like 1 (CHRDL1), Cell Adhesion Molecule L1 Like (CHL1), Cathepsin V (CTSV), L1 Cell Adhesion Molecule (L1CAM) and Delta Like Canonical Notch Ligand 4 (DLL4)) had a statistically significant association with T2D in univariate models. However, following adjustment for group, body mass index (BMI) and age, only ADIPOQ remained statistically significant. In Firth penalised logistic regression adjusted for PMOS status, age and BMI, each 1 SD higher ADIPOQ was associated with lower odds of incident T2D (OR 0.12; 95% CI 0.04–0.31); the association persisted when events were restricted to a common 10-year follow-up horizon, after adjustment for baseline fasting glucose, and after excluding women with baseline fasting glucose ≥ 7.0 mmol/L. Adding ADIPOQ to PMOS status, age and BMI increased the bootstrap optimism-corrected AUC from 0.74 to 0.87. In an independent experimental cohort (PMOS, *n* = 11; controls, *n* = 9, with SOMAscan data), ADIPOQ, neural cell adhesion molecule L1-like protein (CHL1), mast/stem cell growth factor receptor-Kit (KIT), chordin-like protein 1 (CHRDL1) and neural cell adhesion molecule L1 (L1CAM) were measured at 0, 180 and 300 min of 5 h saline and Intralipid infusions and analysed using linear mixed-effects models. Relative to saline, Intralipid lowered L1CAM, CHL1 and KIT at 180 min (differences in change from baseline −0.33, −0.30 and −0.25 log_2_ units; FDR-adjusted *p* ≤ 0.029), with no evidence that responses differed between women with PMOS and controls, and no lipid-specific change in ADIPOQ was detected; only the L1CAM response persisted after excluding turbid or haemolysed samples. Lower adiponectin was the only protein independently associated with incident T2D, whereas L1CAM, and less robustly CHL1 and KIT, responded to acute lipid exposure. The small number of events, differential outcome ascertainment between groups, absence of external validation and the exploratory experimental cohort preclude claims of clinical prediction. These findings support further evaluation of prognostic and stress-responsive protein phenotypes in larger, independently validated cohorts.

## 1. Introduction

Type 2 diabetes (T2D) is projected to affect 1.3 billion people globally by 2050, representing one of the most consequential unmet challenges in chronic disease prevention [1]. Despite the availability of established glycaemic screening tools, traditional risk markers including fasting glucose, haemoglobin A1c (HbA1c), and body mass index (BMI) identify individuals only after substantial metabolic deterioration has occurred, and fail to capture the full spectrum of pathophysiological heterogeneity that precedes overt disease [2]. There is therefore a pressing need for circulating molecular biomarkers that predict incident T2D upstream of glycaemic thresholds and illuminate the biological mechanisms through which risk accumulates [3,4].

High-throughput plasma proteomics offers a particularly promising avenue for this purpose. The foundational candidate selection for the present study was informed by Belongie and colleagues, who applied SOMAscan aptamer-based proteomics to a cohort with impaired glucose tolerance followed prospectively over three years, identifying 43 proteins, spanning prognostic, diagnostic, and combined categories, as candidate monitors of β-cell functional decline and early T2D risk [5]. Subsequently, we demonstrated that, of this 43-protein panel, nine proteins retained significant discrimination between individuals with established T2D of less than 10 years’ duration and non-diabetic controls, most notably adiponectin (ADIPOQ), endocan (ESM1) and Mast/stem cell growth factor receptor-Kit (KIT), all of which were lower in T2D, and that these protein levels were unaffected by acute glucose normalisation, indicating that they reflect stable structural or functional differences rather than transient glycaemic fluctuation [6].

Large-scale proteomic studies have shown that circulating proteins may add prognostic information for incident T2D [7]. However, most studies have been conducted in general-population cohorts without experimental perturbation. Consequently, it remains uncertain whether individual candidate proteins primarily represent stable risk-related phenotypes, dynamic responses to metabolic stress, or both.

PMOS (previously termed polycystic ovary syndrome) affects 5–18% of women of reproductive age and is associated with substantial metabolic heterogeneity [8,9]. Insulin resistance, the defining metabolic feature of PMOS, is present in 65–95% of affected women independent of body mass index (BMI) [10,11]. Prospective UK Biobank data demonstrate increased T2D risk at younger ages, together with differences in HbA1c and liver enzymes [12]. Diagnostic status alone is therefore unlikely to identify all women at greatest metabolic risk, motivating investigation of molecular phenotypes that may refine biological understanding beyond clinical categorisation.

We conducted a two-cohort targeted proteomic study in women with and without PMOS. In Cohort 1, we examined 27 prespecified candidate proteins for association with incident T2D, with correction for multiple testing, and fitted a parsimonious Firth penalised model for the protein retained after covariate and multiplicity adjustment. In an independent experimental cohort, we assessed whether selected candidate proteins changed during controlled lipid versus saline infusion. This design links prospective associations with experimental biological responsiveness, but, of note, the experimental cohort does not constitute validation of T2D prediction.

## 2. Results

### 2.1. Prospective Candidate-Protein Analysis: Candidate-Protein Associations and Penalised Regression

Baseline data were available for 245 women (PMOS, *n* = 148; controls, *n* = 97), of whom 244 had SOMAscan measurements. T2D outcome status at study end was available for 231 women (PMOS, *n* = 135; controls, *n* = 96); 14 women (PMOS, *n* = 13; controls, *n* = 1) were lost to follow-up, although their baseline data were complete. Overall, 25 women developed T2D (PMOS, *n* = 22; controls, *n* = 3). Univariate protein analyses included the 230 women with both outcome and protein data (24 events; one woman who developed T2D had no stored sample). Adjusted analyses included the 221 women with complete age and BMI data (PMOS, *n* = 128; controls, *n* = 93; 20 events) because nine women (four of whom developed T2D) lacked baseline anthropometry. Baseline characteristics of the adjusted analysis sample are summarised in Table 1. Age and height did not differ significantly between groups, whereas women with PMOS had higher mean weight and BMI. Because outcome ascertainment differed between groups (see follow-up data, below), the proportions developing T2D should not be interpreted as directly comparable incidence rates. Median follow-up from baseline to study end was 12.7 years (range 10.2–15.1) and was at least 10 years for every participant; among the 20 cases in the adjusted sample, median time from baseline to diagnosis was 5.2 years (range 0.6–13.6).

Baseline fasting plasma glucose was available for 209 of the 221 women in the adjusted sample (Table 1 does not include HbA1c, which was not measured in Cohort 1. Fasting glucose was higher among women who subsequently developed T2D than among those who did not (median 5.4 [IQR 4.8–6.4] vs. 4.6 [4.4–4.9] mmol/L; Mann–Whitney *p* < 0.001). Five women had a single baseline fasting glucose ≥ 7.0 mmol/L, four of whom subsequently received a diagnosis of T2D, and two further women had impaired fasting glucose (6.1–6.9 mmol/L). Because diabetes had not been diagnosed at enrolment, these women were retained in the primary analysis and examined in sensitivity analyses.

**Figure 1:**
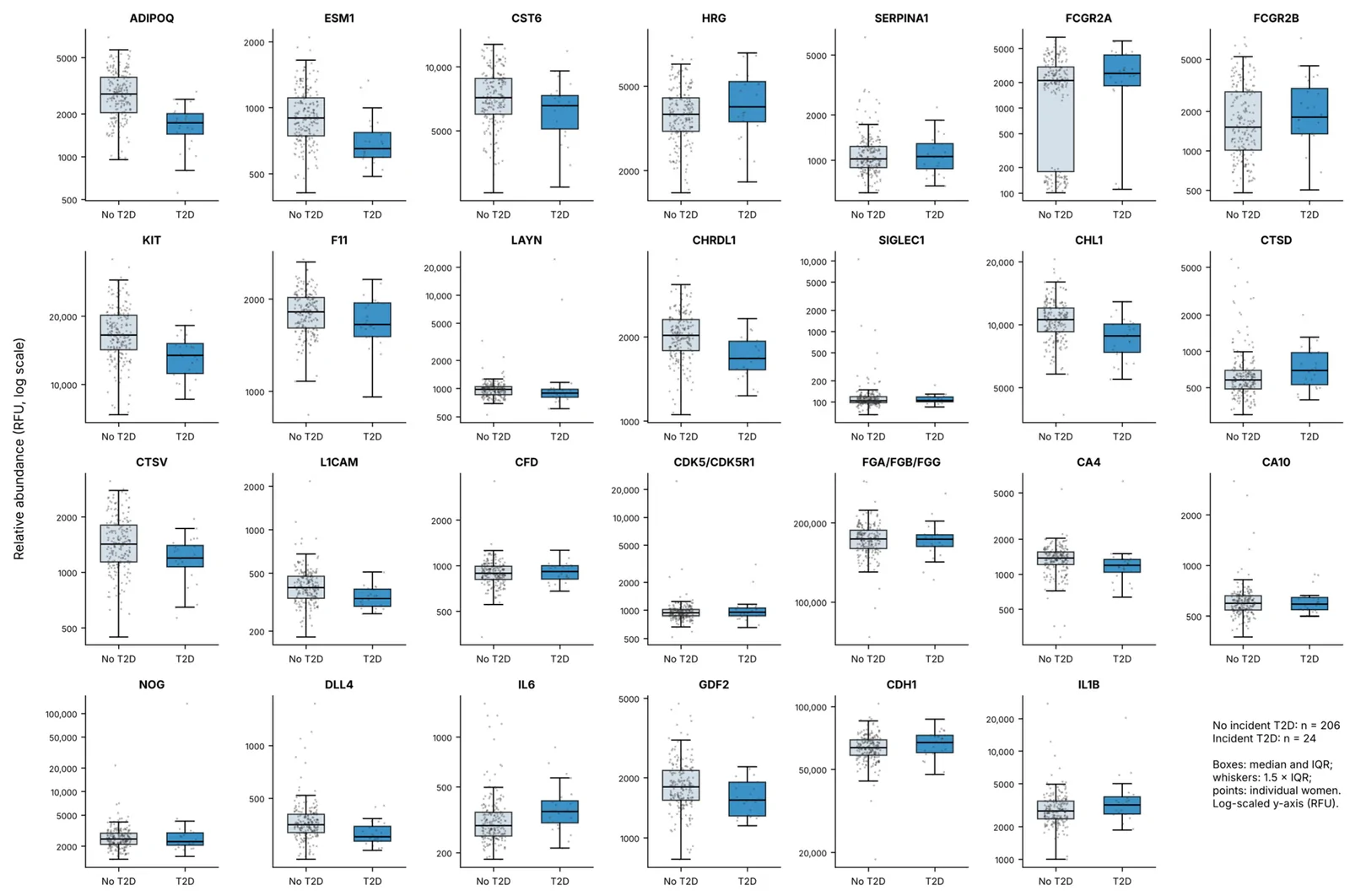
Baseline relative abundance of the 27 candidate proteins with predictive power for the development of type 2 diabetes. Data is presented according to incident type 2 diabetes status. Values are relative aptamer signals in relative fluorescence units (RFUs), not absolute concentrations. Light boxes indicate protein levels of those subjects who did not develop diabetes; dark blue boxes indicate protein levels of those subjects who did develop diabetes.

The 44 biomarkers available were selected and classified based on prior research [5] as prognostic and diagnostic (*n* = 13), prognostic only (*n* = 14), or diagnostic only (*n* = 17). As this analysis is primarily concerned with the predictive power of each biomarker for T2D, only those identified as prognostic or both prognostic and diagnostic were considered for further analysis, resulting in 27 candidate proteins. UniProt accession numbers, SOMAmer SeqIDs, assay dilutions and SOMAscan target names for all 44 proteins are listed in Supplementary Table S1; the fibrinogen measurement (FGA_FGB_FGG) is a single aptamer reagent (SeqId 2796-62_2) targeting the intact fibrinogen complex (UniProt P02671, P02675 and P02679). Summary statistics for each protein, stratified by T2D status, are presented below in Figure 1 and Table 2.

Given the low number of T2D outcomes (24 events among 230 participants with protein data), the analysis initially focused on identifying biomarkers most strongly associated with T2D for consideration in subsequent analyses. Univariate logistic regression models were fitted for each candidate protein, followed by a second set of models adjusted for group (PMOS vs. controls), body mass index (BMI) and age. Adjusted models included the 221 participants with complete age and BMI data (20 events). Models were estimated by maximum likelihood.

The Box–Tidwell test indicated a departure from linearity in the relationship between CA4, IL6 and CTSD and the logit; therefore, these three proteins were log-transformed (natural logarithm). All proteins were standardised using z-scores calculated in the analysed sample to facilitate comparison of the magnitude of associations between biomarkers and T2D. *p*-values were adjusted for multiple comparisons using the Benjamini–Hochberg (BH) procedure. Odds ratios (ORs) with 95% confidence intervals (CIs) and corresponding *p*-values from the univariate and adjusted models are presented in Table 3 and Table 4, respectively.

Following BH adjustment, eight biomarkers (ADIPOQ, ESM1, KIT, CHRDL1, CHL1, CTSV, L1CAM and DLL4) had a statistically significant association with T2D in univariate models. However, following adjustment for group, BMI, and age, only ADIPOQ remained statistically significant.

#### 2.1.1. Logistic Regression–ADIPOQ

After adjustment for multiple comparisons using the BH procedure, only ADIPOQ remained statistically significant. We therefore repeated the analysis using Firth’s penalised logistic regression to further investigate the association between ADIPOQ and type 2 diabetes (T2D).

Given the small number of events (20 events in the adjusted sample), standard logistic regression may produce biased estimates. To address this concern, Firth’s penalised likelihood logistic regression was used. This method helps to reduce the bias caused by rare events in a dataset, producing more reliable odds ratios and *p*-values.

The Firth logistic regression model included Diabetes (Yes/No) as the outcome variable and ADIPOQ, BMI, age and Group as explanatory variables. The 221 participants with complete covariate data were included, of whom 20 developed T2D (approximately five events per model parameter). PMOS status was coded with control as the reference category. Confidence intervals were obtained by profile penalised likelihood and *p*-values from penalised likelihood-ratio tests.

Linearity of continuous predictors with the logit was assessed using Box–Tidwell tests and visual inspection of fitted plots. No predictors indicated a deviation from linearity (*p*-values for all tests >0.05). ADIPOQ remained standardised in this analysis to allow for easier comparisons from previous analyses.

The results of the Firth logistic regression model are presented in Table 5. ADIPOQ remained significantly associated with T2D (*p* < 0.001). Each 1 SD increase in ADIPOQ was associated with 88% lower odds of developing T2D (OR 0.12; 95% CI 0.04–0.31).

Multicollinearity was assessed using variance inflation factors (VIFs), with no evidence of problematic multicollinearity observed (all VIFs < 1.6).

**Figure 2:**
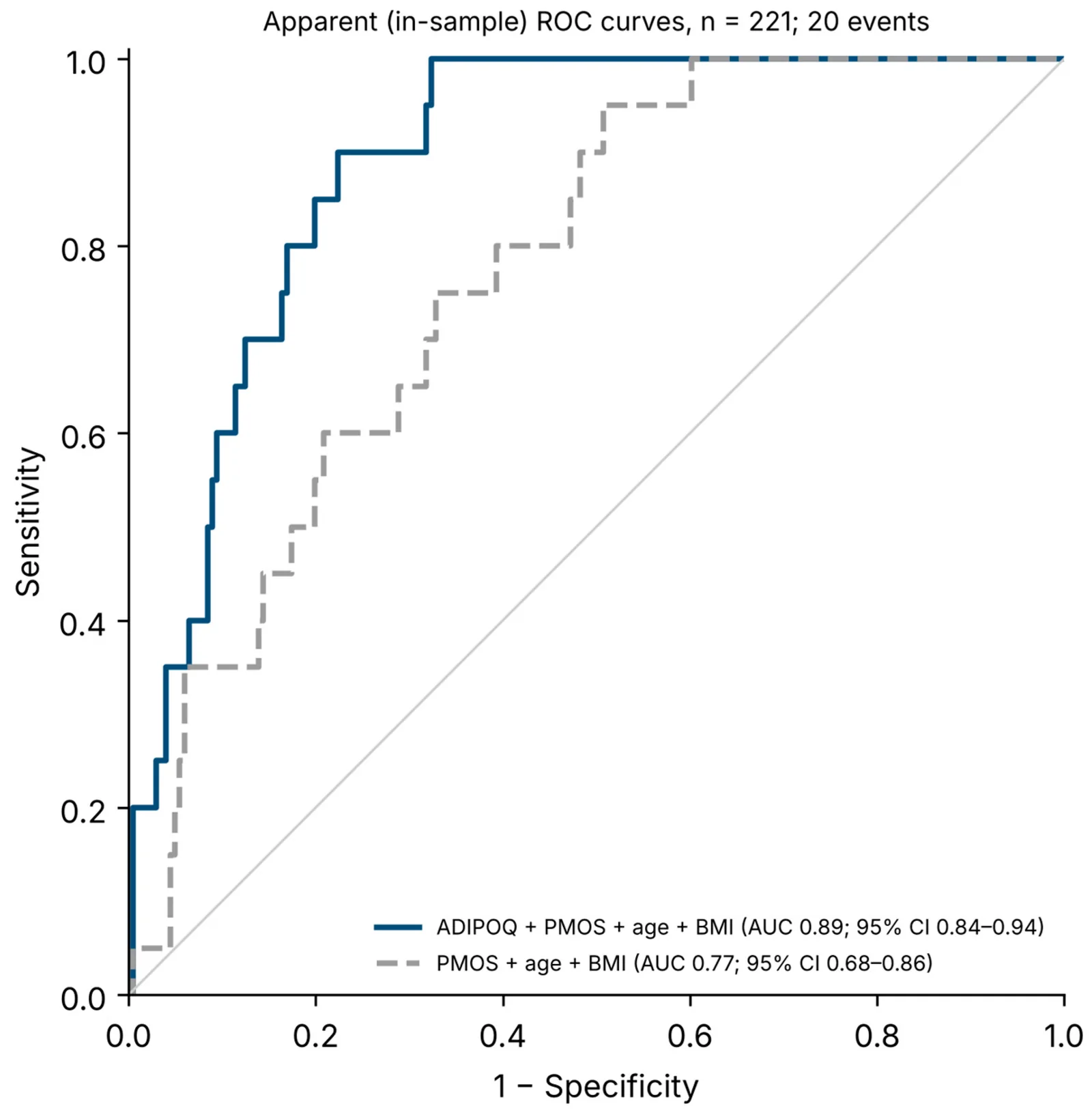
Receiver operating characteristic curve for the Firth penalised logistic regression model including ADIPOQ, PMOS status, age and BMI (apparent, in-sample AUC 0.89; DeLong 95% CI 0.84–0.94).

The ROC curve for the model is shown below in Figure 2. The apparent (in-sample) area under the curve (AUC) was 0.89 (DeLong 95% CI 0.84–0.94), compared with 0.77 (0.68–0.86) for a model containing PMOS status, age and BMI alone. However, given the small number of events in the dataset, even with Firth’s adjustment, there is a risk of overfitting, and the model may not generalise well to new data. The estimate is therefore likely over optimistic and should not be interpreted as clinical predictive performance, but rather as descriptive. It should be noted that due to the nature of our follow-up data, the presence of detection bias is likely in this model. The Firth regression model does not account for unequal follow-up or differences in opportunities to detect diabetes. It should therefore be viewed as exploratory and hypothesis-generating. To further explore our findings, a larger independent validation cohort would be required.

To quantify optimism, both models were internally validated using 500 bootstrap resamples, in which each model was refitted in every resample and evaluated in the original data. The optimism-corrected AUC was 0.87 for the model including ADIPOQ and 0.74 for the clinical model; the difference in AUC was 0.12 (bootstrap 95% CI 0.04–0.19). The optimism-corrected calibration slope was 0.91 and calibration-in-the-large −0.09, indicating modest overfitting. Because the bootstrap did not repeat the selection of ADIPOQ from the 27 candidates, these estimates may remain optimistic, and they should be regarded as descriptive of this sample rather than as validated clinical predictive performance.

A sensitivity analysis was conducted using the same Firth logistic regression model, with baseline fasting glucose added as an additional covariate. (Table 6). We acknowledge that including an additional covariate in a model with a low number of events relative to the number of predictors may affect the stability of the estimates. Furthermore, due to missing baseline fasting glucose data, the analysis included 209 participants, 12 fewer than the primary analysis, including 19 participants who developed T2D (one fewer event). Therefore, this analysis was considered exploratory. ADIPOQ remained significantly associated with T2D in the adjusted model (OR 0.17; 95% CI 0.05–0.48; *p* < 0.001).

#### 2.1.2. Follow-Up Data

An important limitation of this study is the potential for differential ascertainment of diabetes outcomes due to differences in follow-up frequency between groups. Women with PMOS underwent assessments approximately every six months until study end date (26 October 2025), whereas controls were only assessed at baseline and at study end date (26 October 2025). At study end, diabetes status in controls was ascertained from medical records, which provided the calendar year of diagnosis; for all participants, diagnosis dates were recorded to the year only and were assigned to 1 June of that year to calculate time to diagnosis. Diagnoses occurring between scheduled PMOS assessments were captured through review of primary-care and hospital records. These differences may have resulted in unequal opportunities to detect diabetes and uncertainty in the timing of disease onset. Although Firth penalised logistic regression can reduce small-sample estimation bias and mitigate problems associated with sparse data or separation, it does not account for unequal follow-up, interval censoring, or differential ascertainment. In addition, diabetes diagnosis dates were available only by calendar year, limiting the precision with which diagnoses could be assigned to specific follow-up intervals. Accordingly, the observed associations should be interpreted cautiously and viewed as exploratory and hypothesis-generating. Residual bias arising from differences in follow-up and diabetes ascertainment cannot be excluded (Table 7).

Because every participant had at least 10.2 years of follow-up (Table 7), we performed a sensitivity analysis using a common 10-year horizon, in which only diagnoses recorded within 10 years of baseline were counted as events. This horizon was complete for all participants and removes differences in the length of the observation window, although not differences in assessment frequency. Thirteen events (PMOS, *n* = 10; controls, *n* = 3) occurred within this horizon, and ADIPOQ remained inversely associated with T2D (OR 0.15; 95% CI 0.04–0.43; Table 8). Results were also similar after excluding the five women with baseline fasting glucose ≥7.0 mmol/L (OR 0.15; 95% CI 0.04–0.42; 16 events) or the seven women with baseline fasting glucose ≥6.1 mmol/L (OR 0.13; 95% CI 0.04–0.39; 15 events). Because Cohort 1 samples were distributed unevenly by PMOS status across three assay plates, we also adjusted the primary model for plate (OR 0.13; 95% CI 0.04–0.34), standardised ADIPOQ within plate (OR 0.12; 95% CI 0.04–0.32), excluded the two samples that failed the row check (OR 0.12; 95% CI 0.04–0.33; *n* = 219) and excluded each plate in turn (ORs 0.10–0.16); all estimates were consistent with the primary analysis.

### 2.2. Independent Experimental Cohort: CHL1 and L1CAM Show Lipid-Responsive Temporal Dynamics

The demographics of the independent experimental cohort are shown in Table 9.

Linear mixed-effects models assessed five candidate proteins (ADIPOQ, CHL1, KIT, CHRDL1 and L1CAM; all among the eight proteins significant after BH correction in univariate Cohort 1 models) in 115 samples from the 20 participants with SOMAscan data (11 PMOS, nine control). In the revised analysis, one lipid-visit baseline measurement that had been compromised by leakage during the assay, and that had been re-assayed, was excluded; the original analysis had inadvertently included both measurements (Section 4.5.2). No timepoint-by-cohort-by-infusion interaction was statistically significant for any protein (all *p* ≥ 0.057); thus, there was no statistical evidence that the temporal response to lipid versus saline differed between women with PMOS and controls. Fixed-effect tests and all within-condition timepoint contrasts are provided in Supplementary Tables S2 and S3; lipid-versus-saline differences in change from baseline are shown in Table 10, and sensitivity analyses in Supplementary Table S4.

**Figure 3:**
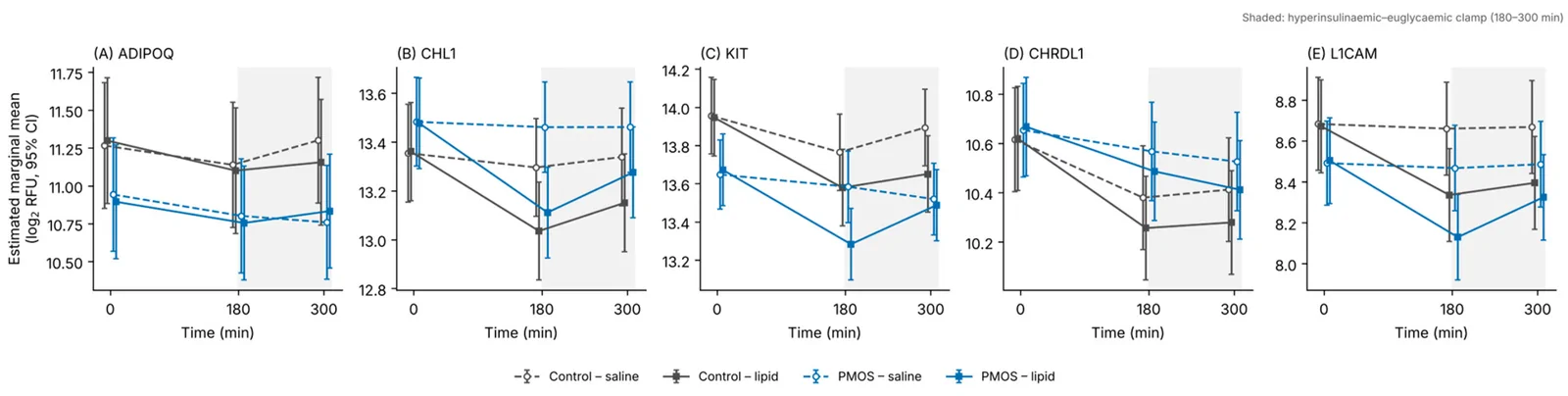
Estimated marginal means for (**A**) adiponectin (ADIPOQ), (**B**) neural cell adhesion molecule L1-like protein (CHL1), (**C**) mast/stem cell growth factor receptor-Kit (KIT), (**D**) chordin-like protein 1 (CHRDL1), and (**E**) neural cell adhesion molecule L1 (L1CAM), by timepoint, PMOS status and infusion condition. Values are estimated marginal means (95% CI) of log_2_ relative fluorescence units (RFUs) from the corrected model (*n* = 115 samples, 20 participants); dashed lines, saline; solid lines, Intralipid. The 300 min sample was obtained during the hyperinsulinaemic–euglycaemic clamp.

ADIPOQ showed a main effect of timepoint (F(2, 85.1) = 11.26; *p* < 0.001), but no significant main effect of cohort or infusion and no timepoint-by-infusion interaction (*p* = 0.85). Relative abundance declined over time under both infusions; after FDR correction, the baseline-to-300 min decline during saline infusion in women with PMOS (−0.18 log_2_ units; FDR-adjusted *p* = 0.025) and the baseline-to-180 min decline during lipid infusion in controls (−0.20; FDR-adjusted *p* = 0.025) were significant. The lipid-versus-saline difference in change from baseline was close to zero (at 180 min, −0.03; 95% CI −0.16 to 0.09; Table 10), so no lipid-specific change in ADIPOQ was detected, although this does not establish stability or equivalence. The numerically lower relative abundance in the PMOS group did not constitute a statistically significant between-group difference (Figure 3A).

CHL1 showed main effects of timepoint (F(2, 85.7) = 12.93; *p* < 0.001) and infusion (F(1, 85.7) = 27.70; *p* < 0.001), and a timepoint-by-infusion interaction (F(2, 85.7) = 8.16; *p* < 0.001). During lipid infusion, CHL1 declined from baseline to 180 min in both controls (−0.33 log_2_ units; FDR-adjusted *p* = 0.001) and women with PMOS (−0.37; FDR-adjusted *p* < 0.001), with smaller declines persisting at 300 min (FDR-adjusted *p* = 0.044 in each group). Relative to saline, the change from baseline was −0.30 (95% CI −0.46 to −0.15) at 180 min and −0.19 (−0.34 to −0.04) at 300 min (Table 10). There was no significant cohort effect or three-way interaction (Figure 3B).

KIT showed main effects of timepoint (F(2, 85.4) = 14.08; *p* < 0.001), infusion (F(1, 85.4) = 9.86; *p* = 0.002) and cohort (F(1, 17.6) = 6.65; *p* = 0.019), with relative abundance 0.27 log_2_ units lower in women with PMOS than in controls (95% CI 0.05–0.48), and a timepoint-by-infusion interaction (F(2, 85.4) = 3.37; *p* = 0.039). During lipid infusion, KIT declined from baseline to 180 min in both controls (−0.37; FDR-adjusted *p* = 0.004) and women with PMOS (−0.39; FDR-adjusted *p* = 0.001); relative to saline, the change from baseline at 180 min was −0.25 (95% CI −0.44 to −0.06; Table 10). The three-way interaction was not significant (*p* = 0.25), so these findings do not demonstrate a PMOS-specific response (Figure 3C).

CHRDL1 showed a main effect of timepoint (F(2, 86.2) = 7.31; *p* = 0.001), with no significant effects of cohort or infusion and no significant interactions. Relative abundance declined gradually under both infusions, and no individual timepoint contrast remained significant after FDR correction. Model residuals for CHRDL1 departed from normality, so these results should be interpreted cautiously (Figure 3D).

L1CAM showed main effects of timepoint (F(2, 85.5) = 11.66; *p* < 0.001) and infusion (F(1, 85.5) = 31.70; *p* < 0.001), and a timepoint-by-infusion interaction (F(2, 85.5) = 9.02; *p* < 0.001). During lipid infusion, L1CAM declined from baseline to 180 min in both controls (−0.34; FDR-adjusted *p* = 0.001) and women with PMOS (−0.37; FDR-adjusted *p* < 0.001) and partially recovered by 300 min, whereas saline trajectories were stable; relative to saline, the change from baseline was −0.33 (95% CI −0.49 to −0.17) at 180 min and −0.22 (−0.38 to −0.06) at 300 min (Table 10). The absence of a significant three-way interaction indicates no evidence that this response differed by PMOS status (Figure 3E).

In sensitivity analyses, results were materially unchanged after adjustment for assay plate and when visit was modelled as a random effect nested within participant (Supplementary Table S5). Because the infusion order was fixed (saline at the first visit and Intralipid at the second), infusion effects cannot be separated from visit effects. Samples recorded as turbid or haemolysed were concentrated in the lipid visit (21 of 38 lipid-visit samples at 180 and 300 min, compared with three of 38 saline-visit samples) and had systematically lower signals. After excluding these samples (*n* = 90), the lipid-associated decline in L1CAM persisted (difference in change from baseline at 180 min −0.21; 95% CI −0.40 to −0.01; timepoint-by-infusion *p* = 0.044), the CHL1 response was attenuated (−0.18; 95% CI −0.36 to 0.00; −0.20 [−0.38 to −0.02] with additional adjustment for plate), and the KIT response was no longer evident (−0.10; 95% CI −0.33 to 0.13). Because this exclusion removes most lipid-exposed samples at 180 min, it may over-correct; nevertheless, interference from lipaemia cannot be excluded for CHL1 and KIT.

## 3. Discussion

This targeted longitudinal study linked prospective protein associations with experimental responses to acute metabolic stress. Following Benjamini–Hochberg adjustment, eight biomarkers (ADIPOQ, ESM1, KIT, CHRDL1, CHL1, CTSV, L1CAM and DLL4) had a statistically significant association with T2D in univariate models. However, following adjustment for group, BMI and age, only ADIPOQ remained statistically significant. Using Firth’s penalised logistic regression, ADIPOQ remained significantly associated with T2D (*p* < 0.001), and this association was robust to a common 10-year follow-up horizon, adjustment for baseline fasting glucose and exclusion of women with diabetic-range baseline fasting glucose.

In Cohort 2, L1CAM, CHL1 and KIT declined during lipid relative to saline infusion, although only the L1CAM response was robust to exclusion of turbid or haemolysed samples, whereas no lipid-specific change in ADIPOQ was detected, and CHRDL1 changed over time without an infusion-specific effect. These findings distinguish a dominant prospective adiponectin signal from exploratory stress-responsive protein phenotypes.

The inverse association between baseline adiponectin and incident T2D is consistent with prospective evidence. A meta-analysis of 34 studies reported a pooled relative risk of 0.53 (95% CI 0.47–0.61) comparing extreme adiponectin tertiles, with attenuation after adjustment for glycaemic markers [13]. Longitudinal trajectory data from the Whitehall II case-cohort study demonstrated that adiponectin declined measurably more than a decade before diabetes diagnosis, independently of concurrent adiposity changes [14]. Mechanistically, adiponectin enhances insulin sensitivity through AdipoR1-mediated AMPK activation in skeletal muscle and AdipoR2-mediated PPARα induction in liver, suppressing hepatic gluconeogenesis and attenuating NF-κB-driven inflammation [15]. The largest cross-ancestry GWAS meta-analysis of adiponectin, encompassing 46,434 individuals, identified 22 associated loci with shared genetic architecture at T2D and metabolic disease trait loci, which is consistent with, but does not by itself establish, a causal role in metabolic risk [16]. The present data reinforce adiponectin as a prospective metabolic-risk marker; although adding ADIPOQ to age, BMI and PMOS status improved internally validated discrimination in this sample, incremental value relative to established glycaemic measures such as HbA1c and oral glucose tolerance testing could not be assessed and requires external validation.

These findings carry particular relevance for women with PMOS, in whom insulin resistance is present in 65–95% and T2D risk is substantially elevated independent of BMI. A large UK Biobank prospective analysis confirmed elevated T2D incidence in PMOS alongside baseline differences in HbA1c and liver enzymes, consistent with intrinsic metabolic dysfunction decoupled from adiposity [12]. The 2023 International Evidence-Based Guideline for PMOS recognises that standard clinical markers are insufficient to capture the full spectrum of metabolic susceptibility in this heterogeneous population [8]. PMOS status was not significantly associated with T2D after adjustment for adiponectin, age and BMI; however, with 20 events and a wide confidence interval, this cannot show that molecular phenotyping supersedes clinical diagnosis. It suggests only that adiponectin captures a component of metabolic susceptibility that is not identical to PMOS status.

CHL1 was associated with incident T2D in univariate analysis, although not after covariate and multiplicity adjustment, and showed a timepoint-by-infusion interaction in Cohort 2. CHL1 is primarily recognised as a neural cell-adhesion molecule involved in cell migration and axon guidance [17,18]. Its response to lipid exposure may indicate sensitivity to acute metabolic perturbation, but direct evidence linking CHL1 to diabetes biology remains limited; the mechanistic interpretation is therefore hypothesis-generating.

KIT showed an inverse association with incident T2D in univariate analysis, which did not survive covariate and multiplicity adjustment, and, in Cohort 2, declined during lipid relative to saline infusion in both women with PMOS and controls, with no evidence of a PMOS-specific response; this response was not evident after excluding turbid or haemolysed samples. KIT relative abundance was also lower in women with PMOS than in controls in Cohort 2. SCF/KIT signalling promotes mitochondrial biogenesis and thermogenesis in brown adipose tissue, and adipose-resident c-kit^+^ progenitors have recently been characterised as committed brown adipocyte precursors with nutrient-responsive differentiation capacity [19]. The lipid-associated KIT decline is compatible with metabolic responsiveness of the KIT pathway; however, as a speculative interpretation, its relationship to adipose progenitor or thermogenic biology remains speculative, particularly given its sensitivity to sample quality.

CHRDL1 was inversely associated with incident T2D in univariate analysis, but this did not survive covariate and multiplicity adjustment. In Cohort 2 it declined over time without an infusion-specific effect. CHRDL1 was recently characterised as a white adipose tissue-specific adipokine that markedly promotes adipogenesis and lipid accumulation through enrichment of lipogenic signalling pathways [20], and additionally stabilises IGFBP3 to modulate IRS1/AKT/mTORC1 signalling during adipocyte differentiation [21]. In Cohort 2, CHRDL1 relative abundance was numerically higher in women with PMOS than in controls, but this difference was not significant. These observations address different comparisons (association with future T2D in Cohort 1 and a PMOS–control difference in Cohort 2) and are therefore not contradictory. Speculatively, CHRDL1 could rise as a compensatory response in insulin-resistant states; this hypothesis requires testing in larger prospective cohorts.

L1CAM demonstrated the most pronounced dynamic profile of all five biomarkers in the independent experimental cohort, with significant effects of timepoint, infusion type and their interaction, and was the only protein whose lipid-associated decline persisted after excluding turbid or haemolysed samples. L1CAM is a transmembrane glycoprotein involved in neuronal migration and axon guidance [22], with reported expression in pancreatic tumour endothelium and pancreatic duct cells [23,24]. Olaniru et al. demonstrated that Schwann cells and endocrine progenitors co-exist, and are thought to be connected by L1CAM-EPHB2 [25]. The strength of L1CAM’s infusion-driven response, together with its univariate association with T2D in the prospective cohort (which did not survive covariate and multiplicity adjustment), identifies it as a metabolically responsive biomarker warranting further investigation in prospective diabetes cohorts. KIT and L1CAM are membrane-associated proteins; the SOMAscan reagent for KIT targets the soluble receptor, and the circulating signals for both proteins most likely reflect shed ectodomains or soluble isoforms. The assay cannot distinguish these molecular species, and no orthogonal immunoassay verification was performed, so tissue-level mechanisms cannot be inferred from these data.

Among participants who developed T2D, the median time from baseline to diagnosis was 5.2 years (range 0.6–13.6). This interval illustrates the long preclinical window during which metabolic risk may evolve. However, women with PMOS were assessed approximately six-monthly, whereas controls were reassessed only at study end, 10.3–15.1 years after baseline; therefore, event proportions and times to diagnosis cannot be compared directly between groups. The analyses are best interpreted as associations with incident T2D across the combined cohort, with PMOS status included as a covariate; the consistency of the ADIPOQ association over a common 10-year horizon provides some reassurance that unequal observation windows do not explain the finding, although differential assessment frequency may still have introduced detection bias.

Strengths of this investigation include the integration of prospective outcome modelling with controlled experimental perturbation, enabling simultaneous assessment of predictive relevance and biological responsiveness, a design rarely employed in proteomic diabetes research alongside the use of Firth penalised regression to mitigate finite-sample bias and FDR correction across timepoint contrasts in the experimental cohort. Key limitations include the modest number of incident T2D events (25 in total, of whom 20 contributed to adjusted models), which constrains statistical power and may have prevented detection of independent signals for other candidate proteins; residual confounding from unmeasured adiposity-related factors cannot be fully excluded; and the predominantly younger female cohort limits generalisability to older or mixed-sex populations. In addition, outcome ascertainment differed between groups (approximately six-monthly assessment in PMOS versus a single end-of-study record review in controls), which may introduce detection bias that adjustment for PMOS status does not remove; diagnosis dates were known only to the calendar year; a few women had diabetic-range fasting glucose at enrolment, raising the possibility of undiagnosed prevalent diabetes, although excluding them did not alter the findings; baseline HbA1c and oral glucose tolerance status were not available; no independent validation cohort for T2D prediction was available; and SOMAscan measurements were not verified with an orthogonal assay. Cohort 2 was small (*n* = 22), lacked statistical power for higher-order interactions, and interrogated biological responsiveness rather than prospective T2D prediction; although the saline arm controls for infusion volume (approximately 450 mL over 5 h) and haemodilution, lipaemia during Intralipid infusion could alter aptamer binding, and this possibility cannot be excluded. Moreover, the infusion order was fixed, so infusion effects cannot be separated from visit effects; and turbid or haemolysed samples, which clustered in the lipid visit, had systematically lower signals, so lipaemia may contribute to the apparent CHL1 and KIT responses, although the L1CAM response persisted after their exclusion, and adjustment for assay plate did not alter the findings. Finally, SomaScan RFU values are relative rather than absolute concentrations, and the findings may not generalise beyond younger women.

## 4. Methods

### 4.1. Study Design and Participants

This study comprised two independent cohorts. Cohort 1 was a prospective longitudinal study of women with PMOS (*n* = 148) and controls without PMOS (*n* = 97) recruited through a metabolic phenotyping programme between October 2010 and October 2015. PMOS was diagnosed using the 2003 Rotterdam criteria (at least two of oligo/anovulation, clinical or biochemical hyperandrogenism, and polycystic ovarian morphology on ultrasonography); controls had normal ovarian function. Exclusion criteria included type 1 diabetes or pre-existing T2D at enrolment, use of insulin-sensitising or hormonal medication, and active inflammatory or systemic illness. Incident T2D was ascertained using World Health Organization criteria: fasting plasma glucose ≥ 7.0 mmol/L, 2 h glucose ≥ 11.1 mmol/L during oral glucose tolerance testing, or HbA1c ≥ 48 mmol/mol. Women with PMOS were assessed approximately six-monthly until study end (26 October 2025), whereas controls were reassessed once, at study end, through review of medical records (10.3–15.1 years after baseline); diagnosis dates were available to the calendar year. Cohort 2 was an independent experimental crossover study of women with PMOS (*n* = 11) and controls (*n* = 9) who were recruited consecutively, each studied during lipid and saline infusion visits with sampling at baseline, 180 min, and 300 min.

### 4.2. Recruitment and Ethical Permission

#### 4.2.1. Cohort 1

Women with PMOS were recruited from the endocrine clinic Hull Royal Infirmary, whereas control participants were recruited by advertisement and underwent clinical and biochemical assessment [26]; controls did not fulfil diagnostic criteria for PMOS. For PMOS diagnosis, the Rotterdam consensus criteria were employed [27]: (1) biochemical (free androgen index (FAI) > 4) and clinical (Ferriman–Gallwey score > 8) hyperandrogenaemia, (2) amenorrhoea or oligomenorrhoea, and (3) transvaginal ultrasound diagnosis of polycystic ovaries. Testing was undertaken to exclude Cushing’s disease, hyperprolactinaemia, non-classical 21-hydroxylase deficiency and androgen-secreting tumours [28]. No other condition or illness was present in participants with PMOS, none of the participants was receiving hormonal therapy, and all had been medication-free (including over-the-counter medication) for at least nine months before enrolment. These criteria were intended to minimise the influence of recognised endocrine or systemic disease on the measured biomarkers. After an overnight fast, waist circumference, height and weight were measured according to WHO guidelines [29], and BMI was calculated as kg/m^2^. Fasting blood samples were centrifuged (3500 g, 15 min), aliquoted and stored at −80 °C. SHBG, insulin and plasma glucose were measured, and HOMA-IR was calculated, as described in Section 4.3.1. Baseline HbA1c were not performed in Cohort 1 but all had an oral glucose tolerance testing, and changes in medication during follow-up were not recorded.

Cohort 1 was conducted in accordance with the Declaration of Helsinki and approved by the Newcastle and North Tyneside Ethics Committee for the PCOS Genetic Bio-bank (REC reference 10/H0906/17; 6 May 2010).

#### 4.2.2. Cohort 2

Twelve women with PMOS were recruited from local endocrine clinics, and 10 healthy control women were recruited through advertisements at Hull University and East Yorkshire Hospital Trust newsletters. Fasting plasma glucose and HbA1c did not differ between controls and women with PMOS (4.89 ± 0.56 vs. 4.94 ± 0.55 mmol/L, and 33 ± 5.6 vs. 34 ± 2.9 mmol/mol, respectively), and no participant had diabetes. The sample size was determined by feasibility and by consistency with previous clamp-based PCOS studies, given the intensive crossover design requiring multiple visits and frequent blood sampling. PMOS was diagnosed using the Rotterdam criteria (i.e., ≥2 of the following: oligomenorrhea, hyperandrogenism or polycystic ovaries on ultrasound as per Cohort 1; other endocrine causes were excluded. All participants with PMOS met all three Rotterdam criteria (Phenotype A).

The inclusion criteria for both groups were: non-smokers, not taking medication, free from concurrent illnesses, and not engaging in regular exercise of at least 90 min of moderate to vigorous intensity per week before the start of the study. Exclusion criteria were a positive pregnancy test, a family history of type 2 diabetes (T2D) and impaired glucose regulation at a screening oral glucose tolerance test. Cohort 2 was approved by the Leeds (Central) Research Ethics Committee (REC reference 10/H1313/44; 2 August 2010) and conducted in accordance with the Declaration of Helsinki.

All participants provided written informed consent before participation.

#### 4.2.3. Protocol

The protocol followed the validated procedures previously described [30]. Briefly, after anthropometry and fasting blood sampling, each participant completed two metabolic studies at baseline with a 5 h infusion of either normal saline (1.5 mL/min) or Intralipid (20% soybean oil, 1.2% egg yolk phospholipids and 2% glycerol; Fresenius Kabi (Runcorn, UK); 1.5 mL/min) in a fixed order, 7 days apart: normal saline at the first study visit and Intralipid at the second. Participants were fasted for 12 h before the infusion was initiated. Three hours into the infusion, a 2 h hyperinsulinemic–euglycemic clamp was initiated using intravenous (i.v.) soluble insulin (Humulin S, Eli Lilly and Co., Indianapolis, IN, USA) at a priming rate of 80 mU/m^2^ surface area/min for 20 min and then 40 mU/m^2^/min till completion. Plasma glucose was clamped at 5.0 mmol/L with a variable infusion rate of 20% dextrose, adjusted to arterialized blood glucose measurements collected every 5 min. The rate of insulin-stimulated glucose disposal (mg/kg/min) (M), a measure of insulin sensitivity, was calculated from the mean of the five 20 min periods from 20 to 120 min during the clamp. Blood samples were collected at baseline and then hourly until study completion; samples at 0, 180 and 300 min were analysed by SOMAscan. Consequently, the 300 min sample was obtained under hyperinsulinaemic–euglycaemic clamp conditions, and changes between 180 and 300 min reflect combined exposure to the infusion and hyperinsulinaemia. Triglyceride, non-esterified fatty acid, glucose and insulin responses to the infusions in this cohort have been reported previously [26]. No other metabolic intervention was undertaken during the visits. Plasma samples were extracted and stored at −80 °C for later analysis. Participants were instructed to maintain their existing dietary and lifestyle habits throughout the study duration.

### 4.3. Biochemical and Hormonal Assays

#### 4.3.1. Metabolic Measures

Fasting blood glucose (FBG) was measured using a Synchron LX20 analyser (Beckman-Coulter, High Wycombe, UK). Serum insulin concentrations were determined by competitive chemiluminescent immunoassay (Immulite 2000, Euro/DPC, Llanberis, UK). Insulin resistance was assessed using the Homeostatic Model Assessment of Insulin Resistance (HOMA-IR), calculated as (insulin × glucose)/22.5 [31]. Glycated haemoglobin (HbA1c) was determined by ion-exchange chromatography (Abbot Diagnostics, Maidenhead, UK). Sex hormone-binding globulin (SHBG) was assessed using an immunometric fluorescence assay (Immulite 2000 analyzer, Euro/DPC, Llanberis, UK). Serum testosterone was quantified using isotope-dilution liquid chromatography tandem mass spectrometry (LC-MS/MS) [32].

#### 4.3.2. Biomarker Quantification

Plasma proteins were quantified using the SOMAscan V3.2 HTS platform (SomaLogic, Boulder, CO, USA), using SOMAscan menu version 1.3k_v01. SOMAscan is an aptamer-based affinity proteomic assay in which chemically modified nucleotide aptamers (SOMAmers) bind target proteins and are quantified by DNA microarray hybridisation [30]; unlike antibody-based immunoassays, including electrochemical immunosensors [31], it reports relative rather than absolute protein abundance, as relative fluorescence units (RFUs). The 1.3k menu comprises 1322 SOMAmer reagents (1306 targeting human proteins, four non-human protein controls and 12 hybridisation controls); for the present analysis, the 44 proteins previously identified by Belongie et al. as candidate monitors of β-cell function [5] were extracted a priori (SOMAmer SeqIDs in Supplementary Table S1). Samples were EDTA plasma stored at −80 °C with no freeze–thaw cycles, and laboratory personnel were blinded to clinical group and outcome. Cohort 1 and Cohort 2 were assayed in separate SOMAscan runs. In Cohort 1, the 244 baseline samples were distributed across three plates (Set 001: 42 PMOS and 42 control; Set 002: 42 and 42; Set 003: 63 and 13), and the proportion of women who subsequently developed T2D did not differ by plate (χ^2^ *p* = 0.69). Hybridisation-control scale factors ranged from 0.80 to 1.72, and 242 of 244 samples passed the row check (all normalisation scale factors within 0.4–2.5). In Cohort 2, 116 samples from 20 of the 22 participants (11 PMOS, 9 control) were assayed on two plates (Set 001, *n* = 84; Set 002, *n* = 31); two participants were not measured due to lack of sample quantity; one sample affected by leakage during equilibration was re-assayed on a third plate; the compromised measurement was excluded from analysis. Fifteen participants had samples on two plates and one on three. All Cohort 2 samples passed the row check (hybridisation scale factors 0.81–1.70), and the five proteins analysed in Cohort 2 passed the calibrator column check. Assay records noted 17 Cohort 2 samples as turbid and 9 as red (haemolysed), predominantly at 180 and 300 min of the Intralipid visit. According to the original assay metadata, raw RFU data underwent sequential intraplate hybridisation normalisation, plate scaling, intraplate median normalisation stratified by sample type, calibration and filtering. The plate-scaling and calibration reference was EDTAPlasma_Tec_Cal_SL16485_8p_1.3k_mm3.4_p3.2. Plate-scale scalar values were 1.601 for Set 002 (PASS) and 2.877 for Set 001 (FLAG). Plate median calibration factors were 1.007 and 1.000 for Sets 002 and 001, respectively, with both passing the plate-median test. Plate-tail percentages were 10.0% for Set 002 and 6.1% for Set 001 and were flagged in the original assay metadata. The assay incorporated 0.005%, 1% and 40% dilution groups. Filtering was performed using SOMAscan menu version 1.3k_v01, with scaleFactor specified as the assay QC pass/fail criterion. The original output reports PASS/FLAG classifications but does not specify a numerical threshold for the scaleFactor criterion. Following the manufacturer’s processing pipeline, normalised RFU values for the prespecified candidate proteins were extracted for the present analysis; no additional investigator-defined filtering thresholds were applied.

### 4.4. Biomarker Selection

Of the 44 plasma proteins available on the SomaScan platform, 27 were prespecified as candidate biomarkers because they had been classified by Belongie et al. as prognostic, diagnostic, or both for incident glucose intolerance (Supplementary Table S1) [5]. All 27 candidates were evaluated for association with incident T2D; the between-group screening step used in the original submission was removed. This selection strategy was targeted and hypothesis-driven rather than an unbiased proteome-wide discovery analysis.

### 4.5. Statistical Analyses

#### 4.5.1. Cohort 1

Univariate logistic regression models were fitted for each of the 27 candidate proteins with incident T2D as the binary outcome, followed by models adjusted for PMOS status (reference: control), age and BMI, estimated by maximum likelihood. Linearity with the log odds was assessed with the Box–Tidwell test; proteins showing non-linearity (CA4, IL6 and CTSD) were natural-log transformed. All proteins were then standardised to z-scores within the analysed sample, so that odds ratios refer to a 1 SD increase. *p*-values were adjusted across the 27 proteins using the Benjamini–Hochberg procedure, separately for univariate and adjusted models. The protein that remained significant after covariate and multiplicity adjustment (ADIPOQ) was analysed further using Firth’s penalised likelihood logistic regression, including PMOS status, age and BMI, with profile penalised likelihood confidence intervals and penalised likelihood-ratio tests. Multicollinearity was assessed using variance inflation factors. Discrimination was summarised by the apparent AUC with DeLong 95% CI and compared with a model containing PMOS status, age and BMI only. Internal validation used 500 bootstrap resamples to estimate optimism-corrected AUC, calibration slope and calibration-in-the-large; the bootstrap did not repeat protein selection. Sensitivity analyses (i) added baseline fasting plasma glucose; (ii) restricted events to diagnoses within a common 10-year horizon from baseline, which was complete for all participants; and excluded women with baseline fasting glucose (iii) ≥7.0 mmol/L or (iv) ≥6.1 mmol/L. Participants with missing outcome or covariate data were excluded from the relevant models (complete-case analysis). No clinical classification threshold was prespecified. Analyses were performed using SAS version 9.4.

#### 4.5.2. Cohort 2

To examine dynamic biomarker responses under controlled metabolic stress, linear mixed-effects models were fitted for each of the five candidate biomarkers (log_2_-transformed) using the model:

log_2_(protein) ~ TP × Cohort × Infusion_Type + (1|Patient_ID)

where TP (timepoint: T0, T180, T300 min), Cohort (PMOS vs. control), and Infusion_Type (lipid vs. saline), along with all two-way and three-way interactions, were included as fixed effects. A random intercept for Patient_ID was included to account for within-individual correlation across repeated measures. Estimated marginal means (EMMs) were derived across the timepoint × cohort × infusion grid, and timepoint contrasts were derived within each cohort-by-infusion condition. To control type I error inflation, the Benjamini–Hochberg false discovery rate (FDR) procedure was applied globally across these timepoint contrasts pooled from the five biomarkers (60 contrasts: three timepoint comparisons in each of four cohort-by-infusion conditions for five proteins); omnibus F-tests were not adjusted across proteins. Fixed effects were assessed using mixed-effects ANOVA with F-tests and Satterthwaite denominator degrees of freedom. Models were fitted by restricted maximum likelihood in R (version 4.3.3) using the lme4, lmerTest (Satterthwaite degrees of freedom) and emmeans packages. All models converged without singular fits; residuals were approximately normal except for CHRDL1. Because saline was always given at the first visit and Intralipid at the second; period and sequence effects could not be estimated separately from infusion effects; the 7-day interval between visits was intended to prevent carry-over. One lipid-visit baseline sample affected by leakage during assay equilibration was excluded and its re-assay retained (115 samples from 20 participants). Lipid-versus-saline differences in change from baseline at 180 and 300 min were estimated as interaction contrasts averaged over cohort, with FDR adjustment across the 10 contrasts. Sensitivity analyses added assay plate as a fixed effect, modelled visit as a random effect nested within participant, and excluded samples recorded as turbid or haemolysed.

## 5. Conclusions

In this targeted two-cohort proteomic study, lower baseline adiponectin was the only protein independently associated with incident T2D after adjustment for covariates and multiple testing, while L1CAM, and less robustly CHL1 and KIT, declined during experimentally induced lipid exposure. KIT and CHRDL1 showed univariate prospective associations that did not survive covariate and multiplicity adjustment. These findings suggest that circulating proteins associated with future T2D may represent biologically distinct phenotypes, including relatively stable risk-associated signals and proteins responsive to acute metabolic perturbation. However, the small number of incident T2D events, differential outcome ascertainment, targeted candidate-selection strategy and exploratory nature of the experimental cohort preclude conclusions regarding clinical prediction. Larger prospective studies with prespecified candidates and independent external validation are required to confirm these findings and determine their potential clinical relevance.

## Figures and Tables

**Table 1:** Participant characteristics in Cohort 1 by polyendocrine metabolic ovarian syndrome (PMOS) status.

Characteristic	PMOS (*n* = 128)	Control (*n* = 93)	Total (*n* = 221)	*p*-Value
Developed diabetes				0.0094 †
No	111 (86.7%)	90 (96.8%)	201 (91.0%)	
Yes	17 (13.3%)	3 (3.2%)	20 (9.0%)	
Time from baseline to T2D diagnosis (years) ‡	7.40 (4.26)	4.91 (4.35)	7.03 (4.25)	— §
Age (years)	27.91 (6.15)	29.44 (6.53)	28.56 (6.34)	0.0804
Height (m)	1.67 (0.07)	1.66 (0.06)	1.67 (0.07)	0.4638
Weight (kg)	95.81 (23.56)	74.17 (18.12)	86.70 (23.93)	<0.0001
BMI (kg/m^2^)	34.11 (7.46)	26.68 (6.45)	30.98 (7.94)	<0.0001
HOMA-IR	3.8 (0.6)	1.6 (0.2)		<0.005
Testosterone (nmol/L)	1.6 (1.0)	1.05 (0.48)		<0.001
SHBG (nmol/L)	42.5 (39.6)	77.5 (78.4)		0.0003

Continuous variables are presented as mean (SD). Analysis sample: women with T2D outcome data and complete age and BMI data. Height, weight and BMI compared using Welch’s *t*-test. BMI, body mass index; PMOS, polyendocrine metabolic ovarian syndrome. † Fisher’s exact test was used because only three controls developed T2D. ‡ Among participants who developed diabetes only (PMOS *n* = 17, control *n* = 3). § Not formally compared; only three controls developed diabetes. HOMA-IR, Homeostatic Model Assessment of Insulin Resistance; SHBG, sex hormone-binding globulin.

**Table 2:** Summary statistics for 27 proteins with predictive power for the development of type 2 diabetes (T2D). ‘No’ indicates subjects who did not develop T2D, and ‘Yes’ indicates the cohort of subjects who did develop T2D during the follow-up period. Analysis sample: *n* = 230 (206 without and 24 with incident T2D). Values are relative fluorescence units (RFUs).

Characteristic	No *n* = 206	Yes *n* = 24
ADIPOQ		
No. obs.	206	24
Mean (SD)	2905 (1085)	1709 (558)
Median (IQR)	2778 (1581)	1734 (572)
Min, Max	954, 6967	558, 2873
ESM1		
No. obs.	206	24
Mean (SD)	949 (280)	722 (211)
Median (IQR)	900 (365)	652 (176)
Min, Max	410, 2099	486, 1331
CST6		
No. obs.	206	24
Mean (SD)	7449 (2149)	6469 (2047)
Median (IQR)	7167 (2820)	6563 (2243)
Min, Max	2551, 13,805	2708, 11,888
HRG		
No. obs.	206	24
Mean (SD)	3854 (1180)	4389 (1589)
Median (IQR)	3690 (1347)	3996 (1858)
Min, Max	1574, 8504	1770, 7166
SERPINA1		
No. obs.	206	24
Mean (SD)	1156 (548)	1128 (370)
Median (IQR)	1022 (343)	1057 (410)
Min, Max	608, 6571	673, 2246
FCGR2A		
No. obs.	206	24
Mean (SD)	2144 (1554)	2782 (1655)
Median (IQR)	2106 (2853)	2549 (2369)
Min, Max	101, 6803	110, 6106
FCGR2B		
No. obs.	206	24
Mean (SD)	2011 (1341)	2319 (1494)
Median (IQR)	1515 (1808)	1811 (1645)
Min, Max	479, 7363	503, 7241
KIT		
No. obs.	206	24
Mean (SD)	17,280 (4741)	13,637 (3070)
Median (IQR)	16,545 (5940)	13,449 (3952)
Min, Max	7361, 35,677	8597, 21,304
F11		
No. obs.	206	24
Mean (SD)	1821 (289)	1712 (308)
Median (IQR)	1815 (418)	1654 (433)
Min, Max	839, 2700	958, 2316
LAYN		
No. obs.	206	24
Mean (SD)	986 (232)	2195 (5005)
Median (IQR)	978 (187)	894 (178)
Min, Max	524, 3256	609, 24,357
CHRDL1		
No. obs.	206	24
Mean (SD)	2062 (421)	1701 (285)
Median (IQR)	2027 (518)	1675 (406)
Min, Max	1054, 3789	1231, 2324
SIGLEC1		
No. obs.	206	24
Mean (SD)	173 (740)	109 (18)
Median (IQR)	104 (21)	105 (18)
Min, Max	66, 10,626	85, 173
CHL1		
No. obs.	206	24
Mean (SD)	10,668 (2347)	8785 (1914)
Median (IQR)	10,576 (2772)	8853 (2710)
Min, Max	3712, 20,578	5478, 12,846
CTSD		
No. obs.	206	24
Mean (SD)	741 (667)	784 (367)
Median (IQR)	578 (208)	692 (444)
Min, Max	297, 5835	395, 2019
CTSV		
No. obs.	206	24
Mean (SD)	1505 (504)	1209 (337)
Median (IQR)	1422 (665)	1197 (327)
Min, Max	446, 3130	568, 1954
L1CAM		
No. obs.	206	24
Mean (SD)	426 (172)	346 (67)
Median (IQR)	398 (141)	333 (94)
Min, Max	182, 2172	262, 510
CFD		
No. obs.	206	24
Mean (SD)	913 (240)	911 (141)
Median (IQR)	891 (180)	915 (182)
Min, Max	340, 3617	679, 1261
CDK5_CDK5R1		
No. obs.	206	24
Mean (SD)	1081 (1655)	1073 (505)
Median (IQR)	943 (149)	954 (181)
Min, Max	518, 24,543	654, 3122
FGA_FGB_FGG		
No. obs.	206	24
Mean (SD)	174,125 (24,843)	172,714 (28,080)
Median (IQR)	174,014 (28,183)	173,602 (17,904)
Min, Max	73,781, 288,460	121,621, 258,983
CA4		
No. obs.	206	24
Mean (SD)	1386 (423)	1356 (1080)
Median (IQR)	1380 (348)	1195 (302)
Min, Max	291, 5366	634, 6300
CA10		
No. obs.	206	24
Mean (SD)	627 (256)	613 (105)
Median (IQR)	596 (117)	588 (99)
Min, Max	376, 3181	499, 884
NOG		
No. obs.	206	24
Mean (SD)	2695 (1552)	7968 (26,782)
Median (IQR)	2472 (811)	2282 (892)
Min, Max	1354, 21,632	1477, 133,658
DLL4		
No. obs.	206	24
Mean (SD)	382 (152)	319 (54)
Median (IQR)	353 (87)	302 (61)
Min, Max	223, 1737	251, 454
IL6		
No. obs.	206	24
Mean (SD)	339 (185)	391 (153)
Median (IQR)	292 (99)	355 (108)
Min, Max	182, 1592	213, 866
GDF2		
No. obs.	206	24
Mean (SD)	1898 (595)	1692 (597)
Median (IQR)	1804 (636)	1548 (617)
Min, Max	780, 4704	1149, 4006
CDH1		
No. obs.	206	24
Mean (SD)	63,854 (9301)	66,346 (9831)
Median (IQR)	63,495 (10,818)	67,230 (12,768)
Min, Max	18,512, 103,319	47,192, 87,002
IL1B		
No. obs.	206	24
Mean (SD)	3135 (2071)	4027 (3648)
Median (IQR)	2798 (1078)	3186 (1153)
Min, Max	996, 27,570	1858, 20,305

**Table 3:** Univariate logistic regression models assessing the association between candidate proteins and T2D. Note: CA4, IL6 and CTSD were log-transformed following evidence of non-linearity from the Box–Tidwell test. All proteins were standardised using z-scores, and thus ORs should be interpreted in terms of 1 SD increase. *n* = 230; 24 events. Models estimated by maximum likelihood.

Protein	OR (95% CI)	*p*-Value	BH Adjusted *p*-Value
ADIPOQ	0.14 (0.06–0.32)	<0.001	<0.001
ESM1	0.27 (0.13–0.55)	<0.001	0.002
KIT	0.34 (0.19–0.62)	<0.001	0.002
CHRDL1	0.33 (0.19–0.58)	<0.001	0.002
CHL1	0.37 (0.21–0.63)	<0.001	0.002
L1CAM	0.25 (0.10–0.63)	0.003	0.015
CTSV	0.47 (0.27–0.81)	0.007	0.023
DLL4	0.17 (0.05–0.58)	0.005	0.017
GDF2	0.63 (0.36–1.10)	0.108	0.199
CST6	0.61 (0.38–0.97)	0.037	0.100
Log(IL6)	1.44 (1.02–2.03)	0.036	0.100
FCGR2A	1.49 (0.98–2.26)	0.063	0.141
F11	0.68 (0.44–1.06)	0.086	0.179
HRG	1.49 (1.00–2.20)	0.048	0.117
FCGR2B	1.23 (0.84–1.80)	0.295	0.379
LAYN	1.84 (0.83–4.11)	0.136	0.216
Log(CA4)	0.73 (0.49–1.08)	0.112	0.199
IL1B	1.25 (0.94–1.67)	0.118	0.199
NOG	1.41 (0.78–2.55)	0.250	0.337
CDH1	1.31 (0.86–1.99)	0.215	0.306
FGA_FGB_FGG	0.94 (0.62–1.45)	0.794	0.874
SIGLEC1	0.07 (0.00–1078.49)	0.585	0.718
CFD	0.99 (0.64–1.53)	0.965	0.983
CA10	0.93 (0.55–1.58)	0.788	0.874
SERPINA1	0.94 (0.58–1.52)	0.809	0.874
Log(CTSD)	1.27 (0.89–1.83)	0.191	0.286
CDK5_CDK5R1	1.00 (0.64–1.54)	0.983	0.983

**Table 4:** Results of adjusted logistic regression models assessing the association between candidate proteins and T2D. Proteins were adjusted for PMOS status, age and BMI. Note: CA4, IL6 and CTSD were log-transformed following evidence of non-linearity from the Box–Tidwell test. All proteins were standardised using z-scores, and thus ORs should be interpreted in terms of 1 SD increase. *n* = 221; 20 events. Models estimated by maximum likelihood; PMOS status reference category: control.

Protein	OR (95% CI)	*p*-Value	BH Adjusted *p*-Value
ADIPOQ	0.10 (0.03–0.30)	<0.001	0.001
ESM1	0.34 (0.15–0.79)	0.012	0.090
FCGR2A	1.85 (1.14–3.00)	0.013	0.090
KIT	0.44 (0.22–0.86)	0.017	0.092
F11	0.66 (0.40–1.09)	0.103	0.232
CHRDL1	0.44 (0.23–0.84)	0.013	0.090
CHL1	0.48 (0.25–0.93)	0.031	0.119
DLL4	0.20 (0.05–0.80)	0.023	0.105
L1CAM	0.32 (0.10–0.97)	0.044	0.132
Log(IL6)	1.43 (0.95–2.15)	0.089	0.232
HRG	1.65 (1.03–2.62)	0.035	0.120
FCGR2B	1.37 (0.89–2.11)	0.148	0.308
IL1B	1.26 (0.91–1.75)	0.163	0.314
CTSV	0.67 (0.37–1.22)	0.191	0.323
NOG	1.51 (0.82–2.78)	0.182	0.323
CDH1	1.49 (0.93–2.40)	0.097	0.232
LAYN	1.71 (0.74–3.97)	0.212	0.337
CST6	0.76 (0.45–1.27)	0.291	0.414
GDF2	0.75 (0.36–1.53)	0.425	0.574
CFD	0.63 (0.28–1.43)	0.266	0.399
Log(CA4)	0.85 (0.55–1.31)	0.468	0.601
Log(CTSD)	1.13 (0.73–1.75)	0.594	0.729
CDK5_CDK5R1	1.09 (0.63–1.90)	0.748	0.777
SIGLEC1	0.20 (0.00–3792.23)	0.746	0.777
CA10	1.08 (0.58–2.00)	0.816	0.816
SERPINA1	0.90 (0.50–1.63)	0.734	0.777
FGA_FGB_FGG	0.88 (0.53–1.46)	0.623	0.731

**Table 5:** Results from Firth logistic regression model. ADIPOQ has been standardised, and so odds ratios should be interpreted relative to a 1 SD increase in ADIPOQ. *n* = 221; 20 events. 95% CIs by profile penalised likelihood; reference category for PMOS status: control.

Variable	OR (95% CI)	*p*-Value
ADIPOQ	0.12 (0.04–0.31)	<0.001
Age	1.04 (0.95–1.14)	0.407
BMI	1.04 (0.97–1.12)	0.283
PMOS vs. Control	1.46 (0.39–6.68)	0.587

**Table 6:** Firth logistic regression model (sensitivity analysis). ADIPOQ has been standardised, and so odds ratios should be interpreted relative to a 1 SD increase in ADIPOQ. *n* = 209; 19 events. 95% CIs by profile penalised likelihood.

Variable	OR (95% CI)	*p*-Value
ADIPOQ	0.17 (0.05–0.48)	<0.001
Age	1.06 (0.96–1.17)	0.227
BMI	1.02 (0.93–1.10)	0.673
PMOS vs. control	2.04 (0.46–12.80)	0.363
Fasting glucose (mmol/L)	3.30 (1.63–6.88)	<0.001

**Table 7:** Average time to diabetes diagnosis and follow-up duration for PMOS vs. control groups. control groups were assessed at baseline and then again at the study end date (26 October 2025) through medical records. PMOS groups were assessed at baseline and approximately six-monthly until the study end date. Analysis sample (*n* = 221). IQR is presented as the interquartile range width. Follow-up duration could not be calculated for one control without a recorded consent date.

	Control *n* = 93	PMOS *n* = 128
Time to diabetes diagnosis (months)		
*n*	3	17
Mean (SD)	59 (52)	89 (51)
Median (IQR)	57 (52)	62 (99)
Min, Max	8, 112	22, 163
Follow-up duration (years)		
*n*	92	128
Mean (SD)	12.54 (0.91)	13.14 (1.23)
Median (IQR)	12.28 (0.94)	12.87 (2.36)
Min, Max	10.26, 15.07	10.17, 14.96

**Table 8:** Sensitivity analyses for the association between ADIPOQ and incident T2D. All models are Firth penalised logistic regression-adjusted for PMOS status, age and BMI (plus fasting glucose or assay plate where stated); ORs per 1 SD increase in ADIPOQ with profile penalised likelihood 95% CIs.

Analysis	*n* (Events)	ADIPOQ OR (95% CI)	*p*-Value
Primary model	221 (20)	0.12 (0.04–0.31)	<0.001
Common 10-year horizon	221 (13)	0.15 (0.04–0.43)	<0.001
Additionally adjusted for baseline fasting glucose	209 (19)	0.17 (0.05–0.48)	<0.001
Excluding baseline fasting glucose ≥ 7.0 mmol/L	216 (16)	0.15 (0.04–0.42)	<0.001
Excluding baseline fasting glucose ≥ 6.1 mmol/L	214 (15)	0.13 (0.04–0.39)	<0.001
Additionally adjusted for assay plate	221 (20)	0.13 (0.04–0.34)	<0.001
Excluding samples failing row check	219 (20)	0.12 (0.04–0.33)	<0.001

**Table 9:** Baseline characteristics of healthy control women and women with polyendocrine metabolic ovarian syndrome.

Parameters	Controls (*n* = 10)	PMOS (*n* = 12)	*p*-Value
Age (years)	25.3 ± 6.5	28.3 ± 6.5	0.290
BMI (kg/m^2^)	26.8 ± 6.5	29.4 ± 5.5	0.320
Waist (cm)	81.1 ± 14.2	98.8 ± 15.8	0.013
Testosterone (nmol/L)	1.05 ± 0.34	1.51 ± 0.69	0.06
FAI	2.1 ± 2.05	6.77 ± 2.90	0.0004
SHBG (nmol/L)	69.6 ± 29.7	26 ± 17.6	0.002
HOMA-IR	1.34 (0.80, 2.13)	2.30 (1.30, 3.90)	0.041

Values are presented as mean ± SD for normally distributed variables and median (interquartile range) for skewed variables. Between-group comparisons were performed using independent t-tests for normally distributed variables, Mann–Whitney U tests for skewed variables and χ^2^ tests for categorical variables. All measurements were obtained under fasting baseline conditions prior to infusion protocols. Abbreviations: BMI, body mass index; FAI, free androgen index; SHBG, sex hormone-binding globulin; HOMA-IR, homeostatic model assessment of insulin resistance; PMOS, polyendocrine metabolic ovarian syndrome.

**Table 10:** Lipid-versus-saline differences in change from baseline in the independent experimental cohort (log_2_ RFU), averaged over women with PMOS and controls. Estimates are interaction contrasts from linear mixed-effects models with a participant random intercept (*n* = 115 samples from 20 participants); negative values indicate a greater decline during Intralipid than during saline. FDR adjustment was applied across the 10 contrasts shown.

Protein	Time from Baseline	Difference (95% CI)	FDR-Adjusted *p*
ADIPOQ	180 min	−0.03 (−0.16 to 0.09)	0.658
ADIPOQ	300 min	−0.03 (−0.16 to 0.10)	0.671
CHL1	180 min	−0.30 (−0.46 to −0.15)	<0.001
CHL1	300 min	−0.19 (−0.34 to −0.04)	0.032
KIT	180 min	−0.25 (−0.44 to −0.06)	0.029
KIT	300 min	−0.14 (−0.34 to 0.05)	0.232
CHRDL1	180 min	−0.11 (−0.38 to 0.16)	0.518
CHRDL1	300 min	−0.13 (−0.40 to 0.14)	0.473
L1CAM	180 min	−0.33 (−0.49 to −0.17)	<0.001
L1CAM	300 min	−0.22 (−0.38 to −0.06)	0.025

## Data Availability

The datasets generated and/or analysed during the current study are not publicly available but are available from the corresponding author on reasonable request. Analysis code, a data dictionary and de-identified analytical data will be provided as Supplementary Material subject to consent and governance requirements.

## Supplementary Materials

The following supporting information can be downloaded at https://www.mdpi.com/article/10.3390/ijms27219894/s1.

## References

[1] Collaborators GBDD (2023). Global, regional, and national burden of diabetes from 1990 to 2021, with projections of prevalence to 2050: A systematic analysis for the Global Burden of Disease Study 2021. Lancet.

[2] Sims E.K., Carr A.L.J., Oram R.A., DiMeglio L.A., Evans-Molina C. (2021). 100 years of insulin: Celebrating the past, present and future of diabetes therapy. Nat. Med..

[3] Yau B., Ghislain J., Kebede M.A., Hughes J., Poitout V. (2025). The role of the beta cell in type 2 diabetes: New findings from the last 5 years. Diabetologia.

[4] Butler A.E., Janson J., Bonner-Weir S., Ritzel R., Rizza R.A., Butler P.C. (2003). Beta-cell deficit and increased beta-cell apoptosis in humans with type 2 diabetes. Diabetes.

[5] Belongie K.J., Ferrannini E., Johnson K., Andrade-Gordon P., Hansen M.K., Petrie J.R. (2017). Identification of novel biomarkers to monitor beta-cell function and enable early detection of type 2 diabetes risk. PLoS ONE.

[6] Moin A.S.M., Sathyapalan T., Atkin S.L., Butler A.E. (2022). Diagnostic and Prognostic Protein Biomarkers of β-Cell Function in Type 2 Diabetes and Their Modulation with Glucose Normalization. Metabolites.

[7] Carrasco-Zanini J., Pietzner M., Koprulu M., Wheeler E., Kerrison N.D., Wareham N.J., Langenberg C. (2024). Proteomic prediction of diverse incident diseases: A machine learning-guided biomarker discovery study using data from a prospective cohort study. Lancet Digit Health.

[8] Teede H.J., Tay C.T., Laven J.J.E., Dokras A., Moran L.J., Piltonen T.T., Costello M.F., Boivin J., Redman L.M., A Boyle J. (2023). Recommendations From the 2023 International Evidence-based Guideline for the Assessment and Management of Polycystic Ovary Syndrome. J. Clin. Endocrinol. Metab..

[9] Joham A.E., Norman R.J., Stener-Victorin E., Legro R.S., Franks S., Moran L.J., Boyle J., Teede H.J. (2022). Polycystic ovary syndrome. Lancet Diabetes Endocrinol..

[10] Cassar S., Misso M.L., Hopkins W.G., Shaw C.S., Teede H.J., Stepto N.K. (2016). Insulin resistance in polycystic ovary syndrome: A systematic review and meta-analysis of euglycaemic-hyperinsulinaemic clamp studies. Hum. Reprod..

[11] Tosi F., Bonora E., Moghetti P. (2017). Insulin resistance in a large cohort of women with polycystic ovary syndrome: A comparison between euglycaemic-hyperinsulinaemic clamp and surrogate indexes. Hum. Reprod..

[12] Henney A.E., Gillespiec C.S., Lai J.Y.M., Schofield P., Riley D.R., Caleyachetty R., Barber T.M., Miras A.D., Dobbie L.J., Hughes D.M. (2025). Risk of Type 2 Diabetes, MASLD and Cardiovascular Disease in People Living with Polycystic Ovary Syndrome. J. Clin. Endocrinol. Metab..

[13] Wang Y., Meng R.W., Kunutsor S.K., Chowdhury R., Yuan J.M., Koh W.P., Pan A. (2018). Plasma adiponectin levels and type 2 diabetes risk: A nested case-control study in a Chinese population and an updated meta-analysis. Sci. Rep..

[14] Tabak A.G., Carstensen M., Witte D.R., Brunner E.J., Shipley M.J., Jokela M., Roden M., Kivimäki M., Herder C. (2012). Adiponectin trajectories before type 2 diabetes diagnosis: Whitehall II study. Diabetes Care.

[15] Begum M., Choubey M., Tirumalasetty M.B., Arbee S., Mohib M.M., Wahiduzzaman M., Mamun M.A., Uddin M.B., Mohiuddin M.S. (2023). Adiponectin: A Promising Target for the Treatment of Diabetes and Its Complications. Life.

[16] Sarsani V., Brotman S.M., Xianyong Y., Fernandes Silva L., Laakso M., Spracklen C.N. (2024). A cross-ancestry genome-wide meta-analysis, fine-mapping, and gene prioritization approach to characterize the genetic architecture of adiponectin. HGG Adv..

[17] Hillenbrand R., Molthagen M., Montag D., Schachner M. (1999). The close homologue of the neural adhesion molecule L1 (CHL1): Patterns of expression and promotion of neurite outgrowth by heterophilic interactions. Eur. J. Neurosci..

[18] Guseva D., Jakovcevski I., Irintchev A., Leshchyns’ka I., Sytnyk V., Ponimaskin E., Schachner M. (2018). Cell Adhesion Molecule Close Homolog of L1 (CHL1) Guides the Regrowth of Regenerating Motor Axons and Regulates Synaptic Coverage of Motor Neurons. Front Mol. Neurosci..

[19] Chen Q., Yu Y., Zhang R., Zhao Q., Yu D., Feng C., Zhou J., Luo M., Yang M., Sun S. (2025). Commitment of adipose-resident c-kit(+) progenitors to brown adipocytes contributes to adipose tissue homeostasis and remodeling. Nat. Commun..

[20] Ahn J., Suh Y., Lee K. (2023). Chordin-like 1, a Novel Adipokine, Markedly Promotes Adipogenesis and Lipid Accumulation. Cells.

[21] Sun H., Wang S., Yang Z., Tian L., Li X., Zhou J., Wang B. (2023). Chordin Like-1 Regulates Osteoblast and Adipocyte Differentiation Through Stabilizing Insulin-Like Growth Factor Binding Protein 3. Stem Cells.

[22] Barry J., Gu Y., Gu C. (2010). Polarized targeting of L1-CAM regulates axonal and dendritic bundling in vitro. Eur. J. Neurosci..

[23] Issa Y., Nummer D., Seibel T., Muerkoster S.S., Koch M., Schmitz-Winnenthal F.H., Galindo L., Weitz J., Beckhove P., Altevogt P. (2009). Enhanced L1CAM expression on pancreatic tumor endothelium mediates selective tumor cell transmigration. J. Mol. Med..

[24] Geismann C., Morscheck M., Koch D., Bergmann F., Ungefroren H., Arlt A., Tsao M.-S., Bachem M.G., Altevogt P., Sipos B. (2009). Up-regulation of L1CAM in pancreatic duct cells is transforming growth factor beta1- and slug-dependent: Role in malignant transformation of pancreatic cancer. Cancer Res..

[25] Olaniru O.E., Kadolsky U., Kannambath S., Vaikkinen H., Fung K., Dhami P., Persaud S.J. (2023). Single-cell transcriptomic and spatial landscapes of the developing human pancreas. Cell Metab..

[26] Butler A.E., Lubbad W., Akbar S., Kilpatrick E.S., Sathyapalan T., Atkin S.L. (2024). A Cross-Sectional Study of Glomerular Hyperfiltration in Polycystic Ovary Syndrome. Int. J. Mol. Sci..

[27] Rotterdam E.-S. (2004). Revised 2003 consensus on diagnostic criteria and long-term health risks related to polycystic ovary syndrome (PCOS). Hum. Reprod..

[28] Legro R.S., Arslanian S.A., Ehrmann D.A., Hoeger K.M., Murad M.H., Pasquali R., Welt C.K. (2013). Diagnosis and Treatment of Polycystic Ovary Syndrome: An Endocrine Society Clinical Practice Guideline. J. Clin. Endocrinol. Metab..

[29] World Health Organization Waist circumference and waist–hip ratio. Proceedings of the Report of a WHO Expert Consultation.

[30] Aye M.M., Kilpatrick E.S., Aburima A., Wraith K.S., Magwenzi S., Spurgeon B., Rigby A.S., Sandeman D., Naseem K.M., Atkin S.L. (2014). Acute hypertriglyceridemia induces platelet hyperactivity that is not attenuated by insulin in polycystic ovary syndrome. J. Am. Heart Assoc..

[31] Matthews D.R., Hosker J.P., Rudenski A.S., Naylor B.A., Treacher D.F., Turner R.C. (1985). Homeostasis model assessment: Insulin resistance and beta-cell function from fasting plasma glucose and insulin concentrations in man. Diabetologia.

[32] Cunningham T.K., Allgar V., Dargham S.R., Kilpatrick E., Sathyapalan T., Maguiness S., Rudin H.R.M., Ghani N.M.A., Latiff A., Atkin S.L. (2019). Association of Vitamin D Metabolites with Embryo Development and Fertilization in Women With and Without PCOS Undergoing Subfertility Treatment. Front. Endocrinol..

