# Supplementary material for: Prospective Proteomic Associations with Incident Type 2 Diabetes and Responses to Metabolic Stress in Women with and Without Polyendocrine Metabolic Ovarian Syndrome
Source: Int J Mol Sci. 2026 Nov 4;27(21):9894. doi: 10.3390/ijms27219894 (PMC13763585; doi:10.3390/ijms27219894)
Supplement: Supplementary file 1 [file ijms-27-09894-s001.zip › ijms-4637531-supplementary.pdf]

Supplementary Table S1. SOMAscan reagents for the 44 β-cell function-associated proteins (Belongie et al., 2017)

Gene symbol	SOMAscan target	UniProt	SOMAmer SeqId	Assay dilution (%)	Category (Belongie et al. 2017)	Analysed as candidate (n = 27)	Column QC check (Cohort 2 run)
ADIPOQ	Adiponectin	Q15848	3554-24_1	0.005	Prognostic and diagnostic	Yes	PASS
ESM1	Endocan	Q9NQ30	3805-16_2	1	Prognostic and diagnostic	Yes	PASS
CST6	Cystatin M	Q15828	3303-23_2	1	Prognostic and diagnostic	Yes	PASS
HRG	HRG	P04196	4996-66_1	0.005	Prognostic and diagnostic	Yes	FLAG
SERPINA1	a1-Antitrypsin	P01009	3580-25_8	0.005	Prognostic and diagnostic	Yes	PASS
FCGR2A	FCG2A	P12318	3309-2_2	1	Prognostic and diagnostic	Yes	PASS
FCGR2B	FCG2B	P31994	3310-62_1	1	Prognostic and diagnostic	Yes	PASS
KIT	SCF sR	P10721	2475-1_3	1	Prognostic and diagnostic	Yes	PASS
F11	Coagulation Factor XI	P03951	2190-55_1	0.005	Prognostic and diagnostic	Yes	PASS
LAYN	Layilin	Q6UX15	2635-61_2	40	Prognostic and diagnostic	Yes	PASS
CHRDL1	CRDL1	Q9BU40	3362-61_2	1	Prognostic and diagnostic	Yes	PASS

SIGLEC1	Sialoadhesin	Q9BZZ2	4464-10_2	40	Prognostic and diagnostic	Yes	PASS
CHL1	CHL1	O00533	3601-54_3	1	Prognostic and diagnostic	Yes	PASS
CTSD	Cathepsin D	P07339	5508-62_3	0.005	Prognostic only	Yes	PASS
CTSV	Cathepsin V	O60911	3364-76_2	40	Prognostic only	Yes	PASS
L1CAM	NCAM-L1	P32004	4246-40_2	1	Prognostic only	Yes	PASS
CFD	Factor D	P00746	2946-52_2	0.005	Prognostic only	Yes	PASS
CDK5 / CDK5R1	CDK5/p35	Q00535 Q15078	3358-51_2	40	Prognostic only	Yes	PASS
FGA / FGB / FGG	Fibrinogen	P02671 P02675 P02679	2796-62_2	0.005	Prognostic only	Yes	PASS
CA4	Carbonic Anhydrase IV	P22748	3177-49_2	40	Prognostic only	Yes	PASS
CA10	Carbonic Anhydrase X	Q9NS85	3289-19_2	40	Prognostic only	Yes	PASS
NOG	Noggin	Q13253	5846-24_3	40	Prognostic only	Yes	PASS
DLL4	DLL4	Q9NR61	3305-6_1	40	Prognostic only	Yes	PASS
IL6	IL-6	P05231	4673-13_2	40	Prognostic only	Yes	PASS
GDF2	GDF2	Q9UK05	4880-21_1	40	Prognostic only	Yes	PASS
CDH1	Cadherin E	P12830	2501-51_3	1	Prognostic only	Yes	PASS
IL1B	IL-1b	P01584	3037-62_1	40	Prognostic only	Yes	PASS

KLK5	kallikrein 5	Q9Y337	3201-49_2	40	Diagnostic only	No	FLAG
KLK4	Kallikrein 4	Q9Y5K2	2833-20_1	40	Diagnostic only	No	PASS
CSF3R	G-CSF-R	Q99062	2719-3_4	40	Diagnostic only	No	FLAG
CDH12	Cadherin-12	P55289	3656-9_1	40	Diagnostic only	No	PASS
IGFBP1	IGFBP-1	P08833	2771-35_2	1	Diagnostic only	No	PASS
ACY1	Aminoacylase-1	Q03154	3343-1_4	1	Diagnostic only	No	PASS
ANGPT4	Angiopoietin-4	Q9Y264	2500-2_3	40	Diagnostic only	No	PASS
OSM	OSM	P13725	2693-20_3	40	Diagnostic only	No	PASS
IGFBP4	IGFBP-4	P22692	2950-57_2	0.005	Diagnostic only	No	FLAG
CA6	Carbonic anhydrase 6	P23280	3352-80_3	1	Diagnostic only	No	PASS
TNFRSF8	CD30	P28908	2605-49_2	40	Diagnostic only	No	PASS
TNFSF8	CD30 Ligand	P32971	3421-54_2	40	Diagnostic only	No	PASS
FCGR3B	FCG3B	O75015	3311-27_1	0.005	Diagnostic only	No	PASS
RTN4R	Nogo Receptor	Q9BZR6	5105-2_3	1	Diagnostic only	No	PASS
LEPR	sLeptin R	P48357	5400-52_3	1	Diagnostic only	No	FLAG
FN1	Fibronectin	P02751	4131-72_2	0.005	Diagnostic only	No	PASS

TNFRSF1B	TNF sR-II	P20333	3152-57_1	1	Diagnostic only	No	PASS

Assay dilution: dilution group (0.005%, 1% or 40% plasma). Column QC check from the Cohort 2 SOMAscan run; the Cohort 1 data file did not include column-level calibration results. FGA / FGB / FGG: a single reagent binding the intact fibrinogen complex (the separate D-dimer reagent was not used); CDK5 / CDK5R1: a single reagent binding the CDK5/p35 complex.

Supplementary Table S2. Fixed-effect tests from linear mixed-effects models in the independent experimental cohort ($\log_2$ RFU ~ timepoint × cohort × infusion + (1 | participant); n = 115 samples, 20 participants; type III F-tests with Satterthwaite degrees of freedom)

Protein	Term	F	Numerator df	Denominator df	p-value
ADIPOQ	Timepoint	11.26	2	85.1	<0.001
ADIPOQ	Cohort	2.1	1	18	0.165
ADIPOQ	Infusion	1.17	1	85.1	0.282
ADIPOQ	Timepoint × Cohort	1.08	2	85.1	0.344
ADIPOQ	Timepoint × Infusion	0.16	2	85.1	0.852
ADIPOQ	Cohort × Infusion	0.68	1	85.1	0.411
ADIPOQ	Timepoint × Cohort × Infusion	2.97	2	85.1	0.057
CHL1	Timepoint	12.93	2	85.7	<0.001
CHL1	Cohort	1.13	1	18.2	0.301
CHL1	Infusion	27.7	1	85.7	<0.001
CHL1	Timepoint × Cohort	0	2	85.7	0.999
CHL1	Timepoint × Infusion	8.16	2	85.7	<0.001
CHL1	Cohort × Infusion	0.28	1	85.7	0.595
CHL1	Timepoint × Cohort × Infusion	0.2	2	85.7	0.815
KIT	Timepoint	14.08	2	85.4	<0.001
KIT	Cohort	6.65	1	17.6	0.019
KIT	Infusion	9.86	1	85.4	0.002
KIT	Timepoint × Cohort	0.15	2	85.4	0.864
KIT	Timepoint × Infusion	3.37	2	85.4	0.039
KIT	Cohort × Infusion	0.3	1	85.4	0.585

KIT	Timepoint × Cohort × Infusion	1.4	2	85.4	0.251
CHRDL1	Timepoint	7.31	2	86.2	0.001
CHRDL1	Cohort	2.69	1	17.7	0.118
CHRDL1	Infusion	1.69	1	86.2	0.197
CHRDL1	Timepoint × Cohort	0.75	2	86.2	0.476
CHRDL1	Timepoint × Infusion	0.55	2	86.2	0.578
CHRDL1	Cohort × Infusion	0.04	1	86.2	0.833
CHRDL1	Timepoint × Cohort × Infusion	0.01	2	86.2	0.992
L1CAM	Timepoint	11.66	2	85.5	<0.001
L1CAM	Cohort	1.62	1	18	0.219
L1CAM	Infusion	31.7	1	85.5	<0.001
L1CAM	Timepoint × Cohort	0.46	2	85.5	0.635
L1CAM	Timepoint × Infusion	9.02	2	85.5	<0.001
L1CAM	Cohort × Infusion	0.39	1	85.5	0.532
L1CAM	Timepoint × Cohort × Infusion	0.32	2	85.5	0.725

Supplementary Table S3. Timepoint contrasts within each cohort-by-infusion condition (60 contrasts; Benjamini–Hochberg FDR across all 60)

Protein	Cohort	Infusion	Contrast (min)	Estimate (log_2)	95% CI	Raw p	FDR-adjusted p
ADIPOQ	Control	Saline	180 - 0	-0.128	−0.259 to 0.003	0.056	0.137
ADIPOQ	Control	Saline	300 - 0	0.034	−0.097 to 0.165	0.604	0.771
ADIPOQ	Control	Saline	300 - 180	0.162	0.031 to 0.293	0.016	0.064
ADIPOQ	PMOS	Saline	180 - 0	-0.141	−0.265 to −0.017	0.026	0.088
ADIPOQ	PMOS	Saline	300 - 0	-0.184	−0.308 to −0.060	0.004	0.025
ADIPOQ	PMOS	Saline	300 - 180	-0.043	−0.167 to 0.082	0.497	0.711
ADIPOQ	Control	Lipid	180 - 0	-0.198	−0.329 to −0.067	0.004	0.025
ADIPOQ	Control	Lipid	300 - 0	-0.143	−0.274 to −0.012	0.033	0.092
ADIPOQ	Control	Lipid	300 - 180	0.055	−0.076 to 0.186	0.406	0.677
ADIPOQ	PMOS	Lipid	180 - 0	-0.14	−0.264 to −0.016	0.028	0.088
ADIPOQ	PMOS	Lipid	300 - 0	-0.061	−0.186 to 0.063	0.328	0.563
ADIPOQ	PMOS	Lipid	300 - 180	0.078	−0.046 to 0.203	0.213	0.387
CHL1	Control	Saline	180 - 0	-0.058	−0.214 to 0.097	0.457	0.711
CHL1	Control	Saline	300 - 0	-0.016	−0.171 to 0.140	0.841	0.914
CHL1	Control	Saline	300 - 180	0.043	−0.113 to 0.198	0.586	0.765
CHL1	PMOS	Saline	180 - 0	-0.022	−0.169 to 0.125	0.766	0.899
CHL1	PMOS	Saline	300 - 0	-0.022	−0.168 to 0.125	0.770	0.899
CHL1	PMOS	Saline	300 - 180	0	−0.147 to 0.148	0.996	0.996
CHL1	Control	Lipid	180 - 0	-0.325	−0.481 to −0.170	<0.001	0.001
CHL1	Control	Lipid	300 - 0	-0.21	−0.365 to −0.054	0.009	0.044
CHL1	Control	Lipid	300 - 180	0.115	−0.040 to 0.271	0.144	0.308
CHL1	PMOS	Lipid	180 - 0	-0.365	−0.513 to −0.217	<0.001	<0.001

CHL1	PMOS	Lipid	300 - 0	-0.201	−0.349 to −0.053	0.008	0.044
CHL1	PMOS	Lipid	300 - 180	0.164	0.016 to 0.312	0.030	0.090
KIT	Control	Saline	180 - 0	-0.191	−0.389 to 0.008	0.059	0.137
KIT	Control	Saline	300 - 0	-0.062	−0.260 to 0.136	0.536	0.731
KIT	Control	Saline	300 - 180	0.129	−0.070 to 0.327	0.200	0.376
KIT	PMOS	Saline	180 - 0	-0.064	−0.250 to 0.122	0.496	0.711
KIT	PMOS	Saline	300 - 0	-0.129	−0.315 to 0.058	0.174	0.342
KIT	PMOS	Saline	300 - 180	-0.065	−0.253 to 0.124	0.497	0.711
KIT	Control	Lipid	180 - 0	-0.367	−0.565 to −0.169	<0.001	0.004
KIT	Control	Lipid	300 - 0	-0.295	−0.493 to −0.097	0.004	0.025
KIT	Control	Lipid	300 - 180	0.072	−0.126 to 0.270	0.473	0.711
KIT	PMOS	Lipid	180 - 0	-0.389	−0.578 to −0.201	<0.001	0.001
KIT	PMOS	Lipid	300 - 0	-0.185	−0.373 to 0.003	0.054	0.137
KIT	PMOS	Lipid	300 - 180	0.204	0.016 to 0.392	0.034	0.092
CHRDL1	Control	Saline	180 - 0	-0.235	−0.514 to 0.043	0.096	0.213
CHRDL1	Control	Saline	300 - 0	-0.203	−0.481 to 0.075	0.151	0.312
CHRDL1	Control	Saline	300 - 180	0.033	−0.246 to 0.311	0.816	0.907
CHRDL1	PMOS	Saline	180 - 0	-0.086	−0.345 to 0.173	0.511	0.713
CHRDL1	PMOS	Saline	300 - 0	-0.128	−0.387 to 0.131	0.328	0.563
CHRDL1	PMOS	Saline	300 - 180	-0.042	−0.306 to 0.222	0.753	0.899
CHRDL1	Control	Lipid	180 - 0	-0.363	−0.641 to −0.085	0.011	0.051
CHRDL1	Control	Lipid	300 - 0	-0.34	−0.619 to −0.062	0.017	0.064
CHRDL1	Control	Lipid	300 - 180	0.023	−0.255 to 0.301	0.871	0.917
CHRDL1	PMOS	Lipid	180 - 0	-0.181	−0.445 to 0.083	0.177	0.342
CHRDL1	PMOS	Lipid	300 - 0	-0.256	−0.519 to 0.008	0.058	0.137
CHRDL1	PMOS	Lipid	300 - 180	-0.075	−0.339 to 0.189	0.575	0.765
L1CAM	Control	Saline	180 - 0	-0.023	−0.185 to 0.139	0.780	0.899

L1CAM	Control	Saline	300 - 0	-0.015	−0.178 to 0.147	0.853	0.914
L1CAM	Control	Saline	300 - 180	0.008	−0.155 to 0.170	0.925	0.957
L1CAM	PMOS	Saline	180 - 0	-0.025	−0.178 to 0.129	0.751	0.899
L1CAM	PMOS	Saline	300 - 0	-0.005	−0.159 to 0.148	0.943	0.959
L1CAM	PMOS	Saline	300 - 180	0.019	−0.135 to 0.173	0.806	0.907
L1CAM	Control	Lipid	180 - 0	-0.336	−0.499 to −0.174	<0.001	0.001
L1CAM	Control	Lipid	300 - 0	-0.277	−0.439 to −0.115	0.001	0.009
L1CAM	Control	Lipid	300 - 180	0.059	−0.103 to 0.222	0.470	0.711
L1CAM	PMOS	Lipid	180 - 0	-0.375	−0.529 to −0.221	<0.001	<0.001
L1CAM	PMOS	Lipid	300 - 0	-0.18	−0.334 to −0.026	0.023	0.080
L1CAM	PMOS	Lipid	300 - 180	0.195	0.041 to 0.349	0.014	0.058

Supplementary Table S4. Lipid-versus-saline differences in change from baseline (interaction contrasts), averaged over cohort and by cohort

Protein	Cohort	Time from baseline	Lipid − saline difference in change (log_2)	95% CI	Raw p	FDR-adjusted p
ADIPOQ	Both (averaged)	180 min	-0.035	−0.162 to 0.093	0.592	0.658
ADIPOQ	Both (averaged)	300 min	-0.027	−0.155 to 0.100	0.671	0.671
ADIPOQ	Control	180 min	-0.07	−0.256 to 0.115	0.453	0.612
ADIPOQ	Control	300 min	-0.177	−0.363 to 0.008	0.061	0.173
ADIPOQ	PMOS	180 min	0.001	−0.175 to 0.177	0.989	0.989
ADIPOQ	PMOS	300 min	0.122	−0.053 to 0.298	0.170	0.283
CHL1	Both (averaged)	180 min	-0.305	−0.456 to −0.154	<0.001	<0.001
CHL1	Both (averaged)	300 min	-0.187	−0.338 to −0.035	0.016	0.032
CHL1	Control	180 min	-0.267	−0.487 to −0.047	0.018	0.072
CHL1	Control	300 min	-0.194	−0.414 to 0.026	0.083	0.200
CHL1	PMOS	180 min	-0.343	−0.551 to −0.135	0.002	0.019
CHL1	PMOS	300 min	-0.179	−0.388 to 0.029	0.090	0.200
KIT	Both (averaged)	180 min	-0.251	−0.444 to −0.058	0.011	0.029
KIT	Both (averaged)	300 min	-0.145	−0.338 to 0.048	0.139	0.232
KIT	Control	180 min	-0.176	−0.457 to 0.104	0.215	0.331
KIT	Control	300 min	-0.233	−0.514 to 0.047	0.102	0.205
KIT	PMOS	180 min	-0.325	−0.590 to −0.061	0.017	0.072
KIT	PMOS	300 min	-0.057	−0.321 to 0.208	0.672	0.707
CHRDL1	Both (averaged)	180 min	-0.111	−0.381 to 0.159	0.414	0.518
CHRDL1	Both (averaged)	300 min	-0.133	−0.402 to 0.137	0.331	0.473
CHRDL1	Control	180 min	-0.128	−0.521 to 0.266	0.520	0.612
CHRDL1	Control	300 min	-0.138	−0.531 to 0.256	0.489	0.612
CHRDL1	PMOS	180 min	-0.095	−0.464 to 0.275	0.611	0.679
CHRDL1	PMOS	300 min	-0.128	−0.497 to 0.242	0.494	0.612

L1CAM	Both (averaged)	180 min	-0.332	−0.490 to −0.174	<0.001	<0.001
L1CAM	Both (averaged)	300 min	-0.218	−0.376 to −0.060	0.007	0.025
L1CAM	Control	180 min	-0.314	−0.543 to −0.084	0.008	0.054
L1CAM	Control	300 min	-0.262	−0.492 to −0.032	0.026	0.086
L1CAM	PMOS	180 min	-0.35	−0.568 to −0.133	0.002	0.019
L1CAM	PMOS	300 min	-0.174	−0.392 to 0.043	0.114	0.208

FDR adjustment applied separately to the 10 averaged contrasts and the 20 cohort-specific contrasts.

Supplementary Table S5. Sensitivity analyses for the independent experimental cohort

Protein	Model	Samples	Timepoint × infusion p	Timepoint × cohort × infusion p	Lipid − saline change at 180 min (95% CI)	p
ADIPOQ	As originally analysed (includes compromised and re-assayed duplicate)	116	0.974	0.278	0.01 (−0.14 to 0.17)	0.893
ADIPOQ	Corrected primary model	115	0.852	0.057	−0.03 (−0.16 to 0.09)	0.592
ADIPOQ	+ assay plate (fixed effect)	115	0.879	0.066	−0.03 (−0.14 to 0.09)	0.657
ADIPOQ	Visit nested within participant (random effect)	115	0.827	0.034	−0.03 (−0.15 to 0.08)	0.561
ADIPOQ	Excluding turbid or haemolysed samples	90	0.648	0.192	0.07 (−0.10 to 0.23)	0.424
ADIPOQ	Excluding turbid or haemolysed samples + assay plate	90	0.853	0.134	0.03 (−0.12 to 0.19)	0.672
CHL1	As originally analysed (includes compromised and re-assayed duplicate)	116	0.016	0.620	−0.26 (−0.43 to −0.08)	0.004
CHL1	Corrected primary model	115	<0.001	0.815	−0.30 (−0.46 to −0.15)	<0.001
CHL1	+ assay plate (fixed effect)	115	<0.001	0.940	−0.30 (−0.44 to −0.17)	<0.001
CHL1	Visit nested within participant (random effect)	115	<0.001	0.807	−0.30 (−0.45 to −0.16)	<0.001
CHL1	Excluding turbid or haemolysed samples	90	0.105	0.902	−0.18 (−0.36 to 0.00)	0.055
CHL1	Excluding turbid or haemolysed samples + assay plate	90	0.059	0.836	−0.20 (−0.38 to −0.02)	0.027
KIT	As originally analysed (includes compromised and re-assayed duplicate)	116	0.684	0.385	−0.12 (−0.42 to 0.18)	0.421
KIT	Corrected primary model	115	0.039	0.251	−0.25 (−0.44 to −0.06)	0.011
KIT	+ assay plate (fixed effect)	115	0.023	0.342	−0.25 (−0.42 to −0.07)	0.007
KIT	Visit nested within participant (random effect)	115	0.036	0.242	−0.25 (−0.44 to −0.06)	0.011
KIT	Excluding turbid or haemolysed samples	90	0.436	0.383	−0.10 (−0.33 to 0.13)	0.385

KIT	Excluding turbid or haemolysed samples + assay plate	90	0.350	0.455	−0.13 (−0.36 to 0.09)	0.242
CHRDL1	As originally analysed (includes compromised and re-assayed duplicate)	116	0.765	0.973	−0.07 (−0.35 to 0.20)	0.590
CHRDL1	Corrected primary model	115	0.578	0.992	−0.11 (−0.38 to 0.16)	0.414
CHRDL1	+ assay plate (fixed effect)	115	0.702	0.971	−0.08 (−0.35 to 0.18)	0.542
CHRDL1	Visit nested within participant (random effect)	115	0.577	0.992	−0.11 (−0.38 to 0.16)	0.414
CHRDL1	Excluding turbid or haemolysed samples	90	0.898	0.134	0.04 (−0.16 to 0.25)	0.672
CHRDL1	Excluding turbid or haemolysed samples + assay plate	90	0.809	0.110	0.04 (−0.15 to 0.23)	0.678
L1CAM	As originally analysed (includes compromised and re-assayed duplicate)	116	<0.001	0.722	−0.33 (−0.49 to −0.18)	<0.001
L1CAM	Corrected primary model	115	<0.001	0.725	−0.33 (−0.49 to −0.17)	<0.001
L1CAM	+ assay plate (fixed effect)	115	<0.001	0.852	−0.32 (−0.47 to −0.17)	<0.001
L1CAM	Visit nested within participant (random effect)	115	<0.001	0.684	−0.33 (−0.48 to −0.19)	<0.001
L1CAM	Excluding turbid or haemolysed samples	90	0.044	0.829	−0.21 (−0.40 to −0.01)	0.036
L1CAM	Excluding turbid or haemolysed samples + assay plate	90	0.040	0.855	−0.22 (−0.41 to −0.03)	0.026
